# Anomalous structural dynamics of minimally frustrated residues in cardiac troponin C triggers hypertrophic cardiomyopathy†

**DOI:** 10.1039/d1sc01886h

**Published:** 2021-04-29

**Authors:** Mayra A. Marques, Maicon Landim-Vieira, Adolfo H. Moraes, Bin Sun, Jamie R. Johnston, Karissa M. Dieseldorff Jones, Elio A. Cino, Michelle S. Parvatiyar, Isela C. Valera, Jerson L. Silva, Vitold E. Galkin, P. Bryant Chase, Peter M. Kekenes-Huskey, Guilherme A. P. de Oliveira, Jose Renato Pinto

**Affiliations:** Institute of Medical Biochemistry Leopoldo de Meis, National Institute of Structural Biology and Bioimaging, National Center of Nuclear Magnetic Resonance Jiri Jonas, Federal University of Rio de Janeiro 373 Carlos Chagas Filho Av, Room: E-10 Rio de Janeiro RJ 21941-902 Brazil gaugusto@bioqmed.ufrj.br +55-21-3938-6756; Department of Biomedical Sciences, Florida State University, College of Medicine 1115 West Call Street, Room: 1370 (lab) – 1350-H (office) Tallahassee FL 32306 USA jose.pinto@med.fsu.edu +1-850-645-0016; Department of Chemistry, Federal University of Minas Gerais Belo Horizonte MG Brazil; Department of Cell and Molecular Physiology, Loyola University Chicago Maywood IL USA; Department of Biochemistry and Immunology, Federal University of Minas Gerais Belo Horizonte MG Brazil; Department of Nutrition, Food and Exercise Sciences, Florida State University Tallahassee FL USA; Department of Physiological Sciences, Eastern Virginia Medical School Norfolk VA USA; Department of Biological Science, Florida State University Tallahassee FL USA

## Abstract

Cardiac TnC (cTnC) is highly conserved among mammals, and genetic variants can result in disease by perturbing Ca^2+^-regulation of myocardial contraction. Here, we report the molecular basis of a human mutation in cTnC's αD-helix (*TNNC1*-p.C84Y) that impacts conformational dynamics of the D/E central-linker and sampling of discrete states in the N-domain, favoring the “primed” state associated with Ca^2+^ binding. We demonstrate cTnC's αD-helix normally functions as a central hub that controls minimally frustrated interactions, maintaining evolutionarily conserved rigidity of the N-domain. αD-helix perturbation remotely alters conformational dynamics of the N-domain, compromising its structural rigidity. Transgenic mice carrying this cTnC mutation exhibit altered dynamics of sarcomere function and hypertrophic cardiomyopathy. Together, our data suggest that disruption of evolutionary conserved molecular frustration networks by a myofilament protein mutation may ultimately compromise contractile performance and trigger hypertrophic cardiomyopathy.

## Abstract 2

Cardiac TnC (cTnC) is highly conserved among mammals, and genetic variants can result in disease by perturbing Ca^2+^-regulation of myocardial contraction.

## Graphical abstract

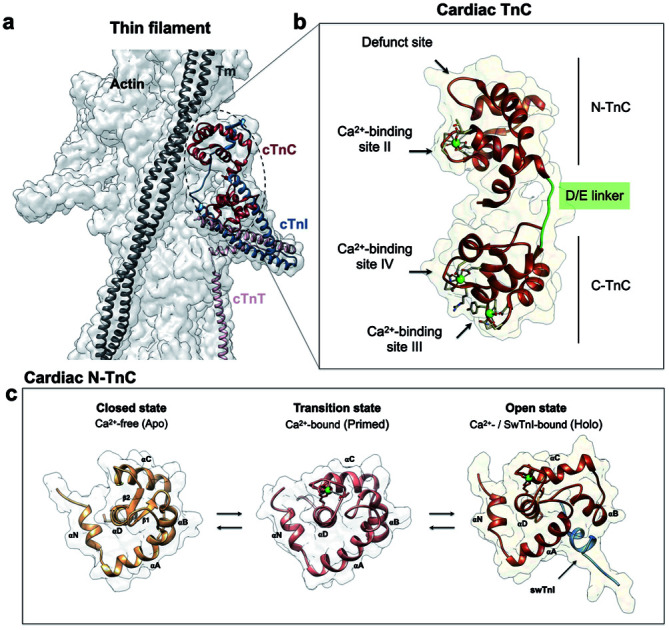

## Introduction

Troponin (Tn) is a sarcomeric protein complex that plays a key role in Ca^2+^-mediated regulation of actomyosin force production in striated muscles. Downstream of an action potential, striated muscle contraction is activated upon an increase in intracellular free Ca^2+^ which promotes binding of Ca^2+^ to troponin C (TnC), triggering molecular rearrangements in thin filament proteins that favor formation of actomyosin cross-bridges.^1^ Regions of proteins or large protein complexes have coevolved to sustain essential communications between distant sites, a molecular process known as allostery.^2^ For instance, an extensive allosteric network maintains physical and dynamic signaling within the thin filament.^3,4^ In the heart, thin filament protein cardiac TnC (cTnC) plays a pivotal role in regulating both the systolic and diastolic phases of the cardiac cycle. Therefore, amino acid variations affecting Ca^2+^-binding affinity of cTnC can instigate cardiac dysfunction and pathological remodeling of the ventricular walls, typically in the form of dilated, hypertrophic, or restrictive cardiomyopathy (DCM, HCM, or RCM, respectively).^5,6^

The overall structure of cTnC consists of two globular domains, each containing EF-hand motifs comprised of helix–loop–helix structures that bind Ca^2+^ and/or Mg^2+^. The two domains of cTnC are connected by an intrinsically disordered structure, the D/E linker. The N and C domains are responsible for Ca^2+^ binding with lower and higher affinities, respectively. cTnC undergoes dynamic processes sampling a range of discrete intermediate states, *i.e.*, the Ca^2+^-free state (apo) or closed conformation, the Ca^2+^-bound state or primed conformation, and the Ca^2+^- and swTnI-bound state or the open, active state. Of particular note, the open state in cTnC is induced when both Ca^2+^ and the switch TnI segment (swTnI, residues 147–163 in cTnI) bind to N-TnC.^7,8^ The sampling of the primed N-TnC to the open (or active) state involves the exposure of a hydrophobic patch to accommodate swTnI_147–163_ which facilitates release of the inhibitory TnI region (iTnI, residues 128–147 in cTnI) from F-actin. These dynamic events that are needed for Ca^2+^ to activate cross-bridge formation are essential for striated muscle contraction,^1,9,10^ and the underlying conformational transitions demonstrate the relevance of evolutionary selection of specific amino acids to function within the organized, native ensemble.

The principle of minimal frustration states that a biomolecule, driven by its polypeptide chain, populates low energy conformation that favors native folds and minimizes non-native contacts. Interestingly, hubs of minimally frustrated interactions are key elements that confer structural rigidity to protein domains.^11^ Whittaker *et al.* presented a link between conformational dynamics and frustration, indicating that non-native interactions often shift the kinetic equilibrium to transient trapping of intermediate species in the higher energy states, known as highly frustrated.^12^ Furthermore, highly frustrated residues are often clustered on protein surfaces and are involved in partner binding, with finely tuned compensation of the entropy and energy changes within the metastable ensemble. Moreover, local violations such as single point mutations, may disrupt the complexity of energy landscapes and interactions of the intermediate ensemble, and thus affect allosteric events and large-scale conformational changes that are particularly important for biomolecule function.^13^

Significant advances in the structural biology field enabled atomistic comprehension of cTn. Through NMR spectroscopy studies, valuable insights into the structure and dynamics of TnC and Tn complex partners have been elucidated.^8,14,15^ Later, Takeda *et al.* provided a high-resolution, X-ray crystal structure of the cTn core domain shedding light on the mechanism of Ca^2+^-regulation in cardiac muscle.^7^ Several MD simulations have focused on the crosstalk between allosteric communications among cTn subunits.^4,16–19^ Despite these achievements, the mechanism of activation of the thin filament remains elusive due to the lack of understanding how relatively small changes in TnC result in large-scale conformational changes within the thin filament upon Ca^2+^ activation. Cryo-EM studies revealed the structure of the whole thin filament at low and high Ca^2+^ levels, but the transitional pathway between the Ca^2+^-free and Ca^2+^-bound states is still inconclusive.^9^ Thus, a comprehensive mechanism of how the cTnC samples a range of discrete states – crucially for activation – is still lacking. Recent cryo-EM analysis of cardiac thin filaments at systolic Ca^2+^ levels^10^ confirmed presence of multiple structural states of the thin filament at physiological Ca^2+^ levels promising to be a valuable tool to bring high resolution NMR data into the context of the whole thin filament.

A study using paramagnetic relaxation enhancement showed that an HCM mutation in cTnC (A8V) induces a more open N-domain conformation in both apo and holo states.^20^ It is not clear what impact pathogenic variants have on this dynamic sampling of open states on muscle contraction and heart remodeling. In the present study, a previously reported, human HCM variant was the focus of a multi-scale investigation from the molecular level—using *in vitro* solution measurements and *in silico* molecular dynamics—to tissue and *in vivo* level studies using a knock-in mouse model. The altered residue (C84Y) is located at the end of the αD-helix and the beginning of the D/E linker in cTnC and increases myofilament Ca^2+^ sensitivity.^21^ The C84Y mutation illustrates how allosteric events drive cTnC sampling among open-closed states and ultimately impact myocardial contraction. Protein contacts exhibit a range of energy degree, or frustration, which controls the kinetics of switching between microstates. Evolution dictates an optimal frustration network to maintain protein function and manage the thermodynamic ensemble. Furthermore, the contact between nonnative interactions can destructively interfere with protein configurational landscapes.^22^ Our data suggest that a single residue change within the core of the minimally frustrated N-TnC hub directly loosens its structural rigidity, which impacts the conformational dynamics of other motifs within cTnC. This leads to altered Ca^2+^ sensitivity of isometric force and altered contractile kinetics, which trigger cardiomyopathy in a mouse model. Because the open state of cTnC precedes muscle contraction, understanding the dynamic processes of this seemingly inactive phase is an essential aspect of heart physiology and disease. We propose that the cTnC N-domain does not simply bind activating Ca^2+^, but responds allosterically to fine-tune myocardial contractility, perturbations in which can be deleterious.

## Results

### Sampling of the cardiac TnC open-like state

**Fig. 1:**
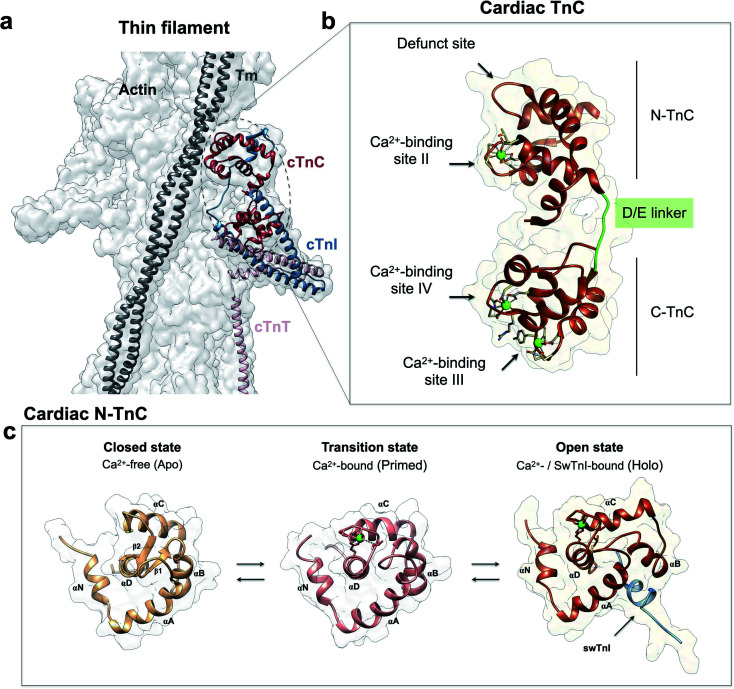
Troponin C is the Ca^2+^ sensor of cardiac muscle. (a) Cardiac thin filament organization. Density maps are from PDB structure 6KN8. (b) Enlargement of the cardiac TnC subunit from the cardiac troponin (Tn) complex (PDB code 1J1E), shown as a ribbon structure. Regulatory and structural domains are N-TnC and C-TnC, respectively. Green spheres represent the 3 Ca^2+^ ions of the Ca^2+^-saturated state. EF-hand residues participating in coordination of Ca^2+^ are shown as gray sticks. The D/E linker is colored green; (c) regulatory domain discrete states prior to contraction. Top view representations of N-TnC in the absence (PDB code 1SPY) or presence of Ca^2+^ (PDB code 1AP4). The open active state of cardiac TnC (PDB code 1MXL) is sampled in the presence of the switch peptide of the TnI subunit (swTnI).

cTnC is essential within the thin filament (Fig. 1a). It responds through precise conformational changes triggered by Ca^2+^ and cTnI-binding (Fig. 1b and c). The discrete conformational states of N-cTnC relevant to this study are shown in (Fig. 1c and ESI Fig. 1a–c†). However, it remains unclear how this sensor tunes the ensemble of conformational states that exist during contraction, and how alterations and preference for specific states can adversely impact heart physiology, leading to disease. To address how cTnC alters its ensemble of conformational states from the closed to the active state, we first designed a Cys-to-Tyr substitution at position 84 (C84Y) of cTnC and characterized in solution whether this substitution can shift the equilibrium toward cTnC open state. To assess the degree of conformational change upon Ca^2+^ binding, the “openness” of the hydrophobic cleft was examined using fluorescence spectroscopy. Fluorescence generated by bis-ANS, a probe for nonpolar cavities of proteins, was measured at *p*Ca 4 and 8, corresponding to the presence or absence of Ca^2+^, respectively. The Ca^2+^-dependent differences in bis-ANS binding indicate that Ca^2+^-induced conformational changes expose more hydrophobic regions in C84Y than unmodified cTnC (ESI Fig. 2a and b†).

**Fig. 2:**
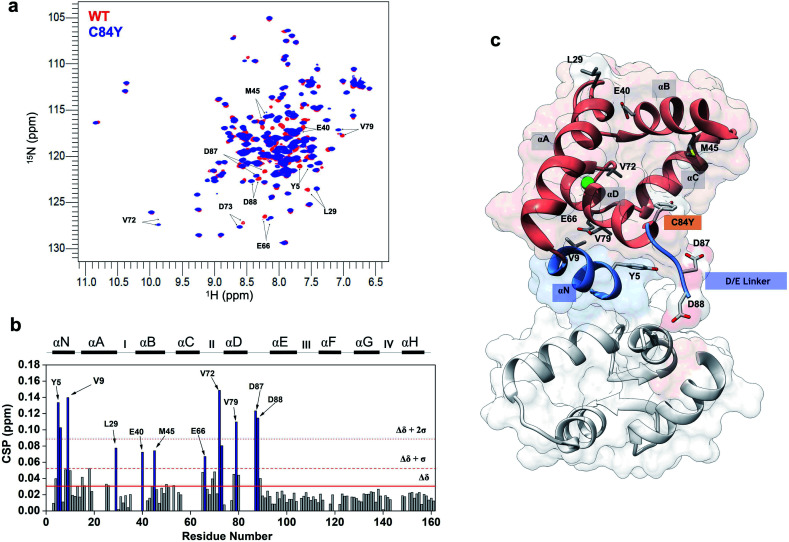
Mutation-induced human cardiac TnC open-like state. (a) Superposition of ^1^H–^15^N HSQC spectra of WT (red) and C84Y (blue) in the primed state. Highlighted residues are those identified as undergoing significant structural changes; (b) chemical shift perturbation (CSP) analysis of C84Y against WT as a function of residue number. Residues deviating from the average values of CSP (Δ*δ*) + 1 S.D. (Δ*δ* + 1*σ*) and + 2 S.D. (Δ*δ* + 2*σ*) are highlighted. Secondary content representation (αN–αH) are depicted as horizontal rectangles in the top. Ca^2+^-binding sites are shown as roman numerals; (c) regulatory (salmon) and structural (gray) cTnC domains show as sticks residues with significant structural changes due to the C84Y substitution. The N-helix (αN) and the D/E linker are colored blue.

To evaluate conformational differences associated with C84Y, we utilized nuclear magnetic resonance (NMR) and performed backbone assignment of C84Y in the Ca^2+^-bound state (Fig. 2a and ESI Fig. 2c†) followed by chemical shift perturbation analysis compared to WT cTnC (Fig. 2b). The most perturbed residues occurred within the αN-helix (αN, residues Y5 and V9), the αB-helix (αB, residues E40 and M45), Ca^2+^-binding site II (residues E66 and V72), and the D/E linker (residues D87 and D88). Superposition of N-cTnC structures in the primed and open active state revealed that conformational changes mostly occurred within the N-, B-, and C-helices, consistent with what was captured experimentally for C84Y by chemical shift analysis (Fig. 2 and ESI Fig. 1c†). These results suggest that C84Y shifts the native cTnC conformation to an average-weighted distribution of states that are more open compared to WT.

In the absence of the swTnI peptide, which stabilizes the open state, we hypothesized that C84Y presents lower structural rigidity in solution. Thermostability measurements derived from circular dichroism (CD) and Kratky plots from chemical-induced unfolding of small-angle X-ray scattering (SAXS) illustrated that C84Y has lower structural stability compared to WT cTnC in solution (ESI Fig. 3a–e†). Melting temperature (*T*_m_) values corresponding to the unfolding processes of the C- (1st) and then the N-domain (2nd) in the Ca^2+^-bound (primed) state, revealed decreased values for both domains upon C84Y substitution (ESI Fig. 3a–c†). In contrast to WT cTnC in the Ca^2+^-free (apo) state, C84Y exhibited one transition, indicative of cooperativity loss upon thermal unfolding (ESI Fig. 3a–c†).

Kratky plots derived from SAXS data are often used to infer subtle changes in the folding behavior of proteins against treatments. A Kratky plot with a bell-shaped distribution is indicative of a well-folded protein.^23^ By adding increasing amounts of denaturant to WT and C84Y in the Ca^2+^-free and -bound (primed) states, we noticed that, in contrast to WT, less denaturant was required to reduce the bell shaped Kratky plots for C84Y in either state (ESI Fig. 3d and e†). This shows that C84Y is less structurally stable than WT when subjected to chemical-induced unfolding. Because temperature is an important physical property impacting the range of conformational changes that can occur in proteins, we performed a local temperature variation analysis by ^1^H–^15^N heteronuclear single quantum correlation (HSQC) experiments (ESI Fig. 3f†). By measuring the temperature coefficients for the chemical shifts of the amide hydrogens (^1^H) as a function of residue position in the primary sequence (ESI Fig. 3g†), we uncovered several distinctions between WT and C84Y (ESI Fig. 3h–t†). Residues of C84Y located within the αN-helix (V9), the αB-helix (E40 and K43), the D/E central-linker (D87), and the αE-helix (L100 and N107) responded differently to temperature variations. This suggests that helices αN, αB, and the D/E linker of C84Y are prone to distinct conformational changes.

Because the C84Y substitution induces a less stable, open-like conformation in cTnC, we expected that it would affect its binding properties to cTnI. To test this hypothesis, we used ^1^H–^15^N HSQC spectroscopy to titrate two previously characterized consensus cTnI peptides referred to as the inhibitory peptide, iTnI_128–147_, and the switch peptide, swTnI_147–163_ (ESI Fig. 4a†). Surprisingly, regardless of whether the C84Y substitution was present, the peptides bound with equal affinity to cTnC (ESI Fig. 4b–g†) and with no apparent changes in their binding affinities for C84Y compared to WT (ESI Fig. 4h and i†). However, distinct regions in the D/E linker were perturbed when comparing WT *vs.* C84Y such as E96 and L97 for WT *vs.* E94, E95 and L97 for C84Y (ESI Fig. 4c†). This indicates that the lower structural rigidity and the open-like ensemble of states triggered by the C84Y substitution do not affect the binding properties between cTnC and cTnI consensus peptides, but rather are more likely explained by changes in conformational dynamics within cTnC (ESI Fig. 5†). Hence, we evaluated C84Y dynamics at different timescales.

Altogether, the C84Y substitution: (i) induces greater hydrophobic exposure of normally buried residues compared to WT; (ii) induces structural changes in several regions that are equally disturbed as the protein samples the active state; (iii) modulates temperature-induced conformational changes of some residues in these regions; (iv) reduces overall structural integrity of the protein; and (v) does not affect the binding of cTnI consensus peptides. Thus, our findings are suggestive that the C84Y substitution causes cTnC to sample an ensemble of states similar to the open state in solution.

### Conformational dynamics of minimally frustrated residues in cTnC

**Fig. 3:**
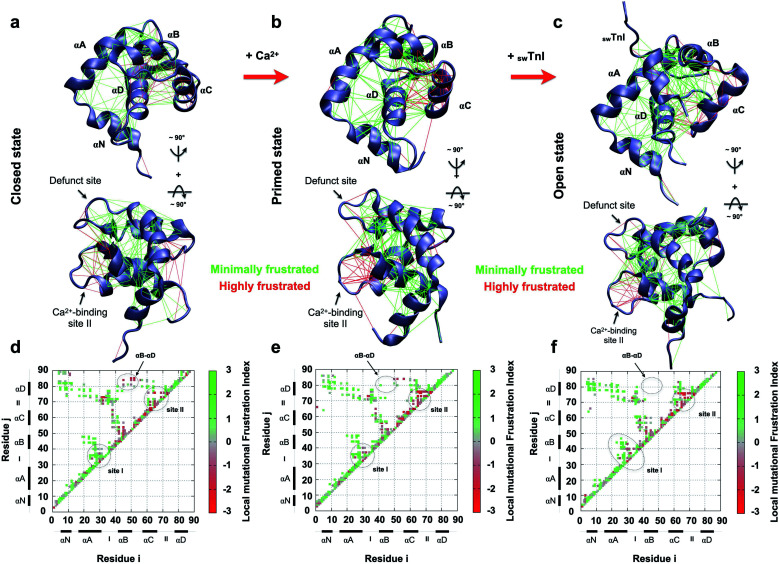
Frustration analysis of discrete human cardiac TnC N-domain conformational states. (a–c) Tertiary frustration patterns of the closed, primed and open active states (PBD codes: 1SPY, 1AP4 and 1MXL), respectively, in two different views. Cα contact pairs within a distance of 5 Å are shown as red lines for highly frustrated interactions and green lines for minimally frustrated pairs; (d–f) contact maps for each studied case in (a–c), respectively. The color scale indicates the local mutational frustration index (see Methods).

To understand how the non-evolutionary, pathological introduction of tyrosine at position 84 in cTnC-C84Y impacts the funneled energy landscape driving protein folding, we examined how energetics were impacted within N-TnC structure. We performed a theoretical analysis of how extensively non-conservative substitutions shift the energetics of the protein and report a local mutational frustration index (see Methods). Then, we assessed the frustration levels of native cTnC contact pairs compared to the conformational dynamics of the mutation-induced ensemble of states. We performed this approach for the three previously solved, discrete conformational states of cardiac N-TnC (Fig. 3a–f). We discovered a net of minimally frustrated interactions governed by helix αD. This is an important finding because minimally frustrated nets confer structural rigidity to protein domains. Thus, it is expected that the pathological C84Y substitution impacts the conformational dynamics of other cTnC motifs. It is interesting to note that interactions within helix αC and those between helix αB and the B/C loop are highly frustrated, and these are the segments that participate in large scale motions toward the open state (Fig. 3a–c).

In contrast to interactions within the defunct Ca^2+^ binding site I of cTnC, those within Ca^2+^-binding site II are highly frustrated (Fig. 3d–f). More interesting is the observation that in their open state, skeletal and cardiac TnC isoforms present segments with different frustration profiles (ESI Fig. 6†). For example, while sTnC presents highly frustrated interactions within both functional Ca^2+^-binding sites of the N-domain (ESI Fig. 6a and c†), the defunct site I of the cardiac isoform does not (ESI Fig. 6b and d†). Additionally, contact pairs within the cardiac D/E linker are minimally frustrated when compared to the corresponding skeletal segment (ESI Fig. 6c and d†). These findings may provide insights to explain how the evolutionary sequence diverged to acquire specialized functions in these two distinct muscle types.

**Fig. 4:**
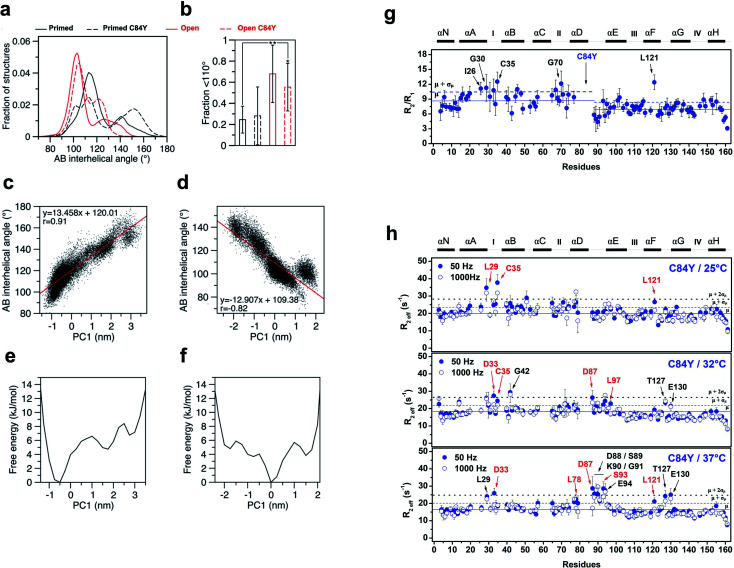
MD simulations and experimental dynamics. (a) AB interhelical angle distributions; (b) fractions under 110° (*t*-test: *, *p* = 0.015; **, *p* = 0.005); (c) comparison of the first principal component (PC1) projections with AB interhelical angles for WT primed; (d) comparison of the first principal component (PC1) projections with AB interhelical angles for WT open; (e) PC1-based free energy landscapes for WT primed; (f) PC1-based free energy landscapes for WT open; (g) *R*_2_/*R*_1_ relaxation ratio per residue for C84Y. Solid and dashed blue lines represent the *R*_2_/*R*_1_ avg. values among residues for C84Y and 1 S.D. from avg., respectively. Ca^2+^-binding sites and secondary structure elements are shown along the top.^59^-^15^N NOE data per residue. Residues with *R*_2_/*R*_1_ values > avg. + 1 S.D. are indicated by arrows. (h) Quantitative analysis of exchange dynamics in C84Y. (A) *R*_2eff_ values for each residue obtained by CPMG relaxation dispersion in the holo state at 50 Hz (filled circles) and 1000 Hz (open circles). Upper panel, 25 °C; middle panel, 32 °C; lower panel, 37 °C. Residues experiencing intermediate exchange are indicated by red arrows.

We conducted additional simulations using a computational model that calculates the time-dependent behavior of fluctuations and conformational changes of proteins. To assess how C84Y altered sampling of the open state, we performed microsecond molecular dynamics (MD) simulations using the primed and open active discrete states of cardiac N-TnC and calculated the AB interhelical angles for each system (see Methods). The findings suggest that, independent of C84Y substitution, when cardiac N-TnC is bound to the swTnI peptide, the open conformation (AB interhelical angle < 110°) is significantly more favored (Fig. 4a and b), with major populations displaying interhelical angles ∼100°. In agreement with previous results (Fig. 2 and ESI Fig. 2a and b†), the MD simulations indicate that in the primed state, the C84Y substitution may increase the population of open structures relative to WT, although the difference was not statistically significant (Fig. 4a and b). The WT primed and open state trajectories were analyzed by principal component analysis (PCA). The first two principal components described 51% and 54% of the variance for the primed and open active systems, respectively, with the first principal component (PC1) accounting for 30% of the variance in both cases. Free energy landscapes based on the PC1 projections show a flatter distribution for the open active compared to the primed state, indicating that N-cTnC may be able to sample open conformations more readily when bound to the swTnI peptide (Fig. 4e and f). Strong correlations were observed between the PC1 projections and the AB interhelical angles (Fig. 4c, d and ESI Fig. 7a and b†). In an attempt to correlate our experimental evidence in isolated cTnC with theoretical models, we investigated whether the C84Y TnC impacts intra-TnC interactions for the intact troponin core domain (Tn, PDB code 1J1E) and thin filament–troponin (TF–Tn) structures (calcium-free state, PDB code 6KN7; calcium-bound state, PDB code 6KN8). We report the contact map analyses based on our simulation data (ESI Fig. 7c†). Data show significant contacts between helices N, A (residues M1-D25) and D (residues V72-M85), and between B (residues T38-M47) and C (residue Q50-T71) of the N-terminal domain. Comparable interactions are shown for residues L97-D113 and T129-E161 of the C-terminal domain. These interactions contribute to the packing of helical bundles in the TnC globular domains. Upon binding Ca^2+^ and swTnI peptide, interactions between G49-D65 with D73-S89 evident in the TF–Tn Ca^2+^-free state are disrupted in the intact Tn and TF–Tn Ca^2+^-bound states (ESI Fig. 7c†). These regions correspond to the two helices flanking the EF-hand Ca^2+^ binding site II in N-terminal domain of TnC; disruption of this interaction is commonplace in Ca^2+^-binding proteins and contributes to their high affinities for ions.^24^ Unique to the C84Y structures, we observe that the mutation enhances the intra-contacts between helix C (residues P52-T71) and helix D (residues D73-M85) in the intact Tn (ESI Fig. 7c†). This is consistent with our observations in the isolated TnC structure that C84Y alters interactions with helix D, which were shown to contribute to the protein's minimally frustrated interactions. Further, we observed similar AB-interhelical angles for the C84Y variant and WT structures, albeit those distributions may be slightly and insignificantly shifted toward higher angles relative to the WT equivalents (ESI Fig. 7d†). This is consistent with the root mean square fluctuations (RMSFs) that demonstrate comparable trends for WT and C84Y cTnC (ESI Fig. 7e†). In summary, the intact and TF–Tn simulations do not strongly evidence differences in the dynamics of the N-terminal domain. This is not surprising, in that our data for the isolated structure here and a previous study^25^ suggest AB opening dynamics occur at a microsecond timescale, which were not practicable for the intact and TF–Tn simulations.

To experimentally probe the conformational dynamics of C84Y and correlate the findings with the frustration levels of structures within cTnC obtained from MD simulations, we assessed the dynamics of C84Y cTnC in the Ca^2+^-bound (primed) state in different timescale regimens. We carried out nuclear spin relaxation experiments to measure the *R*_1_ (longitudinal) and *R*_2_ (transverse) rates derived from internuclear ^15^N–^1^H vectors of the protein backbone, that directly inform about protein diffusional properties within the fast, pico-to nano-second timescale. We observed that the N- and C-domains of C84Y cTnC present distinctly different diffusional properties in solution, with different correlation times and slightly lower *R*_2_/*R*_1_ values for the C-domain compared to the N-domain. This indicates that the tumbling of the two domains in solution occurs in a manner consistent with independent motion despite physical connection *via* the D/E linker. A group of residues located within the defunct site I (I26, G30, and C35) revealed *R*_2_/*R*_1_ ratios that were one standard deviation above the average values of the N-domain indicative of fast conformational exchange dynamics (Fig. 4g).

To assess the intermediate exchange dynamics of C84Y, we performed Carr-Purcell-Meiboom-Gill (CPMG) relaxation dispersion measurements at 25, 32, and 37 °C. These experiments are sensitive to millisecond exchange dynamics, which have been shown to be relevant for functional aspects of biomolecules.^26^ By observing the effective decay of magnetization *R*_2eff_, which reports on the effective transverse relaxation rate, with refocusing pulses of 50 and 1000 Hz, we pinpointed two classes of dynamic regimens: (i) fast-exchange motions in the microsecond timescale and (ii) intermediate exchange motions in the millisecond timescale. Residues undergoing fast-exchange motions are qualitatively observed as those presenting nearly identical values of *R*_2eff_ for the 50 and 1000 Hz refocusing pulses one standard deviation above the average of residues. In contrast, intermediate exchange residues correspond to those in which *R*_2eff_ of refocusing pulses are separated by a decay and values are one standard deviation above the average of all residues. At physiological temperature, we revealed that C84Y samples fast- and intermediate-exchange motions within the defunct site (residues L29, D33, and C35) and the D/E linker (residues D87, D88, S89, K90, S93, E94, and L97) (Fig. 4h and ESI Fig. 8†). The dynamic properties experimentally captured in these NMR measurements are of fundamental relevance to the mechanism by which this Ca^2+^ sensor is allosterically tuned during contraction. The Cys-to-Tyr substitution within the minimally frustrated hub of N-TnC loosens its structural rigidity and, because of that, the conformational dynamics of other TnC motifs including the D/E-linker and cTnC's defunct site I are allosterically altered (Fig. 4h and ESI Fig. 8†). Remarkably, these motifs have diverged through evolution to acquire specialized functions in skeletal and cardiac muscle.

### Generation and *in vivo* characterization of a KI-*Tnnc1*-p.C84Y mouse model

**Fig. 5:**
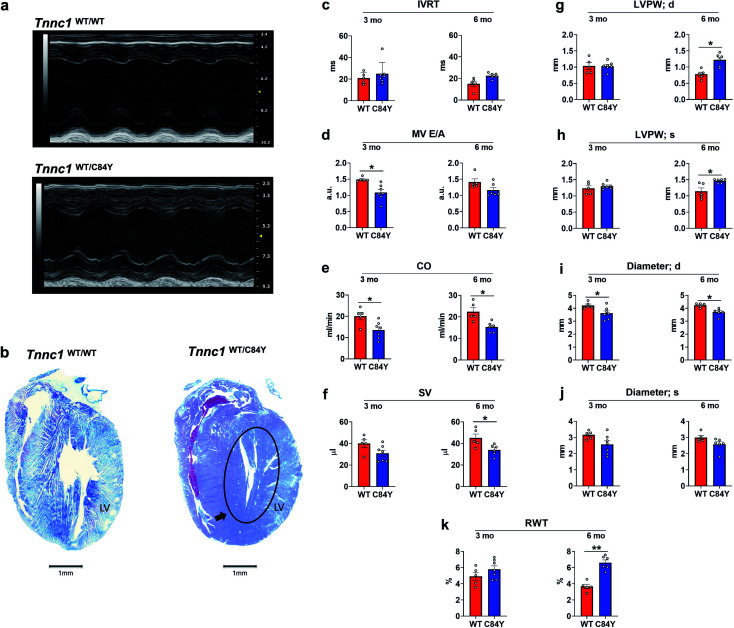
Knock-in mouse model bearing variant *Tnnc1*^WT/C84Y^ recapitulates hypertrophic cardiomyopathy. (a) Echocardiography reveals differences in cardiac structure and function in 3 month-old knock-in *Tnnc1*^WT/C84Y^ mice; (b) representative tissue slices from *Tnnc1*^WT/WT^ and *Tnnc1*^WT/C84Y^ mouse hearts stained with Masson's trichrome. Representative, longitudinally sectioned, stained hearts exhibit morphology of advanced cardiac disease in 14 month-old *Tnnc1*^WT/C84Y^ but not *Tnnc1*^WT/WT^ mouse hearts. Note the extensive hypertrophy of the left ventricle (LV) in *Tnnc1*^WT/C84Y^ heart (circled area). (c–k) M-mode and pulsed wave Doppler measurements were analyzed in both the *Tnnc1*^WT/WT^ and *Tnnc1*^WT/C84Y^ mice at 3 and 6 months of age. (c)IVRT – isovolumetric relaxation time; (d) MV E/A – mitral valve early peak flow velocity – atrial peak flow velocity ratio; (e) CO – cardiac output; (f) SV – stroke volume; (g) LVPW – left ventricular posterior wall thickness during diastole (d) or; (h) systole (s); (i) diameter during diastole (d) or; (j) systole (s); (k) relative ventricular wall thickness; ANOVA with least significant difference post-hoc test was performed to determine significance. *p < 0.05; **p < 0.001 WT *vs.* C84Y within age time point. The heart rate was statistically significant lower in *Tnnc1*^WT/C84Y^ compared to *Tnnc1*^WT/WT^ (3 months, 504 *vs.* 450 bpm and 6 months 501 *vs.* 452 bpm, ESI Table 1†).

To determine whether the C84Y allele associated with HCM in humans could be recapitulated in a small rodent model, we employed a gene-targeting strategy to generate KI-*Tnnc1*-p.C84Y mice (ESI Fig. 9,† see Methods). Interestingly, homozygous (*Tnnc1*^C84Y/C84Y^) mice were not obtained, indicating that the presence of two C84Y alleles is embryonically lethal. Therefore, all of our experiments were carried out using heterozygous (*Tnnc1*^WT/C84Y^) mice. Mice heterozygous for the C84Y allele (*Tnnc1*^WT/C84Y^) displayed *in vivo* functional and morphological features consistent with cardiomyopathy by 3 months of age (Fig. 5 and ESI Table 1†). Further evidence of disease in *Tnnc1*^WT/C84Y^ mice was increased myocardial fibrosis by 8–9 months of age compared to WT (ESI Fig. 10†). Not only were indices of left ventricular hypertrophy increased in the 6 month-old *Tnnc1*^WT/C84Y^ mice but overall, there was a significant increase in relative ventricle weight (RWT)% when considering multiple ventricular thickness measurements in these mice at this age. M-mode echocardiography revealed narrowing of the ventricular lumen by reduced LV diameter values in 3 month and 6 month-old *Tnnc1*^WT/C84Y^ mouse hearts compared to age-matched WT controls. Doppler mitral flow measurements in *Tnnc1*^WT/C84Y^ hearts provided evidence of diastolic dysfunction as early as 3 months of age, with significantly elevated isovolumetric contraction time (IVCT) (ms), an early indicator of asymptomatic diastolic dysfunction. The 3 month-old mice had increased index values for (IVRT + IVCT)/ET of 1.04 for WT hearts *versus* 0.786 for *Tnnc1*^WT/C84Y^ hearts, whereas by 6 months of age the *Tnnc1*^WT/C84Y^ mice had increased isovolumetric relaxation time (IVRT). Both ages of *Tnnc1*^WT/C84Y^ mice had decreased mitral valve early peak flow velocity/atrial peak flow velocity (MV E/A) (Fig. 5). Additional informative echocardiographic parameters are reported in ESI Table 1.† Histopathological analysis of *Tnnc1*^WT/C84Y^ mice at 14 months of age indicated advanced pathological remodeling with gross morphological features indicated by overt left ventricular hypertrophy of the myocardium compared to age-matched WT control mice (Fig. 5b). In concordance with human clinical data^21^ these cumulative results suggest that expression of a single C84Y allele at the *Tnnc1* locus is sufficient to cause cardiomyopathy in mice.

### *Tnnc1* ^WT/C84Y^ mice display HCM-related abnormalities in cardiac muscle mechanics

**Fig. 6:**
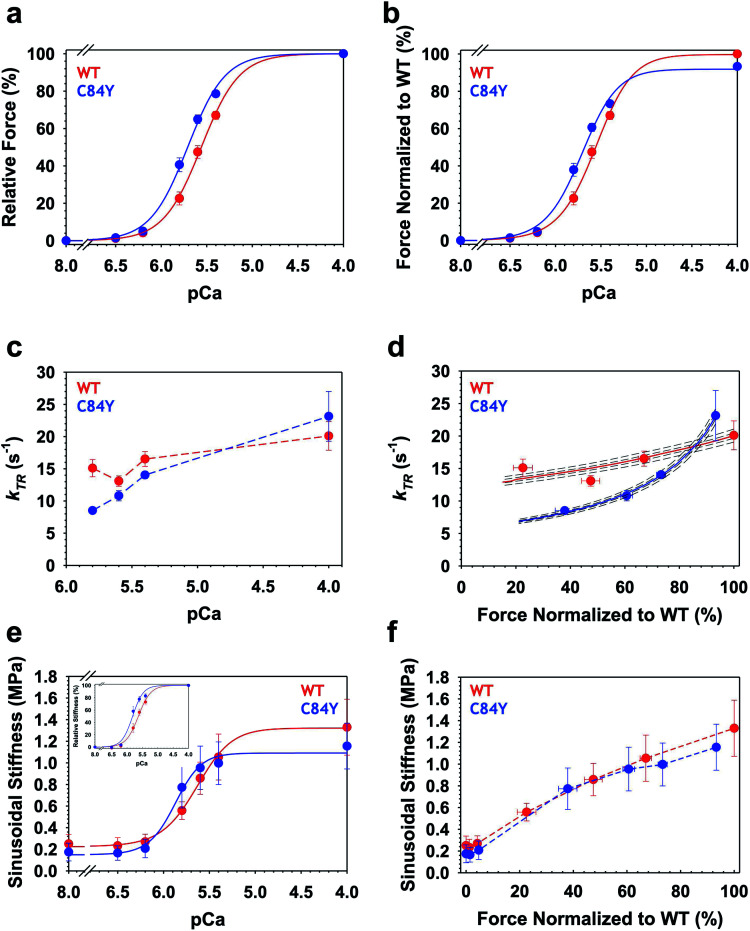
Ca^2+^-dependence of steady-state isometric tension, kinetics of tension redevelopment (*k*_TR_) and sinusoidal stiffness (SS) in knock-in *Tnnc1*^WT/C84Y^ CMPs. (a) Relative steady-state isometric force as a function of *p*Ca. Force values were normalized to the maximum force generated by each individual CMP. (b) Normalized steady-state isometric force as a function of *p*Ca. The C84Y force values (blue) were normalized to average maximum force produced by WT (red). (c) Ca^2+^-dependence of *k*_TR_. Dashed lines were drawn by eye. (d) *k*_TR_ as a function of normalized steady-state isometric force. The force values were normalized to the maximum steady-state isometric force generated by WT. Both solid and dashed lines represent solutions obtained from a 3-state model describing force-*k*_TR_ relationships: solid lines represent the best fit parameters (*f*, *g*, *k*_OFF_) and *k*_ON_^63^ and dotted lines represent solutions when all four parameters were either increased by 1 or 3% (upper lines) or decreased by 1 or 3% (lower lines). (e) Ca^2+^-dependence of sinusoidal stiffness. (f) Sinusoidal stiffness as a function of normalized steady-state isometric force. Force values were normalized to the maximum steady-state isometric force generated by WT. Dashed lines were drawn by eye. Data are shown as mean ± S.E.M. (*n* = 5–6). Best fit parameter estimates from non-linear least square regression on the Hill equation (panels a, b and e) and the 3-state model for force-*k*_TR_ relationships (panel d) are summarized in ESI Tables 2 and 3,† respectively.

To determine whether myofilament dysfunction underlies the cardiac dysfunction observed *in vivo*, we carried out muscle mechanics experiments using permeabilized cardiac muscle preparations (CMPs) isolated from *Tnnc1*^WT/C84Y^ or *Tnnc1*^WT/WT^ mice at 6 months of age (ESI Fig. 11†). In line with the paradigm for most sarcomeric HCM variants, we observed significantly increased myofilament Ca^2+^ sensitivity of steady-state, isometric tension production for *Tnnc1*^WT/C84Y^ CMPs (*p*Ca_50_ = 5.72 ± 0.02) with a leftward shift of 0.16 *p*Ca units compared to *Tnnc1*^WT/WT^ control CMPs (*p*Ca_50_ = 5.56 ± 0.02). There were no significant differences in maximum isometric tension (*F*_max_) or cooperativity of thin filament activation (*n*_Hill_) (Fig. 6a and b, and ESI Table 2†). Measurements of maximal sinusoidal stiffness (SS _max_), which is used as an index of the overall number of cross-bridges, revealed no significant difference between from *Tnnc1*^WT/C84Y^ (01.15 ± 0.21) and *Tnnc1*^WT/WT^ (01.33 ± 0.25) (Fig. 6e and f, ESI Table 2†).

Intriguingly, Ca^2+^ activation of the kinetics of isometric tension redevelopment (*k*_TR_) for *Tnnc1*^WT/C84Y^ CMPs was more curvilinear than that obtained for WT (Fig. 6d). This was mainly due to slower *k*_TR_ for *Tnnc1*^WT/C84Y^ CMPs at submaximal Ca^2+^-activation because there was no significant difference in maximum *k*_TR_ (Fig. 6c and d, and ESI Table 2†). Computational modeling based on a 3-state model of striated muscle contraction (see Methods) that accounts for the differences in Ca^2+^ sensitivity of isometric force (ESI Table 3†) suggests that *Tnnc1*^WT/C84Y^ CMPs exhibit ∼2-fold faster rate of cross-bridge attachment (*f*) and ∼2-fold slower rate of cross-bridge detachment (*g*) (Fig. 6d, and ESI Table 3†). The more curvilinear relation between force and *k*_TR_ for *Tnnc1*^WT/C84Y^ CMPs (Fig. 6d) results from faster regulatory unit dynamics (specifically *k*_OFF_) (ESI Table 3†) combined with these changes in *f* and *g*. Taken together, we conclude that the *Tnnc1*-p.C84Y variant increases the magnitude while decreasing the kinetics of isometric tension production at submaximal Ca^2+^ activation in cardiac muscle, with no measurable impact on maximal values of the parameters measured or the overall number of cross-bridges.

## Discussion

Despite our in-depth understanding of many aspects governing muscle physiology, a number of key topics remain vaguely delineated. Of particular significance for normal and pathophysiology of the heart, it is still not precisely known exactly how Ca^2+^ ions trigger contraction—especially not the atomistic details that dictate overall function—and how this process is modulated by single amino acid substitutions in cTnC such as C84Y that are associated with cardiomyopathy. Here, using a translational investigation, we (i) fashioned a system in which conformational dynamics reflect the intermolecular sampling of the open active state by cTnC in solution, (ii) investigated how the system behaves in terms of Ca^2+^ sensitivity and kinetics of force production within the context of the thin filament unit and the sarcomere, and (iii) determined the systemic effects in mouse models. We demonstrated how this cardiac sensor responds allosterically to Ca^2+^ binding when sampling conformations that favor the open state of the N-domain. To further define the consequences of the disturbance generated by the C84Y substitution, we also provide functional readouts of cardiac muscle function in a newly developed knock-in mouse model.

To experimentally investigate the cardiomyopathy variant, we introduced within the regulatory core (helix αD) of cTnC a Tyr side chain at position 84 in a bacterially expressed, recombinant protein for *in vitro*, solution experiments and also, *via* modification of the genome, in our mouse model. Because helix αD is the central hub connecting all other helices of the regulatory domain, and Tyr has a bulky side chain, we expected that C84Y would shift the conformational equilibrium toward a more open conformation. Our initial experiments revealed that C84Y cTnC exhibits Ca^2+^-induced conformational changes that lead to greater hydrophobic exposure in the N-domain, and structural changes in regions there are equally disturbed during open state sampling. It is worth mentioning that upon Ca^2+^ binding, the cTnC WT structure is known to be less affected than sTnC and binding of swTnI is required for the major structural displacement of BC helices toward the open conformation of cTnC. In this scenario, the NAD helices are mostly unaffected with a RMSD of 1.05 Å.^8^ Our MD simulations revealed an AB interhelical angle of ∼100° which was similar for both the WT and C84Y open states bound to swTnI. This agrees with previous measurements of 102 ± 5°,^8^ which supports the finding that the swTnI peptide stabilizes the open state regardless of whether C84Y is present when using NcTnC only. This is consistent with our swTnI peptide titrations as most of the structural changes observed were similar for WT and the C84Y (ESI Fig. 4†). However, chemical shift changes were observed for both N-terminal and D/E linker backbone atoms upon swTnI binding using full cTnC. In the primed state, C84Y revealed a dual distribution of trajectories with AB interhelical average angles of ∼110° and ∼150°, indicating that the substitution favors the ensemble of more open conformations (Fig. 4a). This is in line with greater hydrophobic exposure and the structural changes within helices αN and αB (Fig. 2 and ESI Fig. 2 a and b†). Because highly frustrated αB-αD tertiary interactions are progressively lost upon Ca^2+^-binding to the N-domain due to large motions toward the open state (Fig. 3a–f), this favoring of open conformations also explains why C84Y in either the Ca^2+^-free (apo) or Ca^2+^-bound (primed) state exhibits less structural rigidity than WT, as evidenced by temperature- and chemical-induced unfolding at lower temperatures or denaturant concentrations, respectively (ESI Fig. 3†).

In sTnC, αN stabilizes the protein and participates in the Ca^2+^-switching mechanism.^27^ Further, the structural C-terminal domain is also impacted,^28^ an observation that was subsequently demonstrated to occur in cTnC.^29^ In fact, cTnC can assume bent conformations in solution, which is partially due to the flexible nature of its central helix, the D/E linker.^30,31^ The flexibility of this linker is of pivotal importance to the association of both domains, and the dynamic repertoire that TnC adopts during activation of cardiac muscle contraction. Studies aimed toward elucidating the functionality of this linker utilized truncations of the skTnC linker, which impaired its flexibility and regulation of the actomyosin ATPase.^32–34^ In calmodulin (CaM), transient interdomain association leads to compact states and is similarly modulated by the length and rigidity of the central linker.^35^ In the context of TnC's binding partner TnI, when cTnI_1–80_ is bound to cardiac C-TnC, the D/E linker binds the inhibitory cTnI peptide, iTnI_129–149_.^36^ This is aligned with our titrations using iTnI_128–147_ that affect a cluster of residues located at the beginning of αE (E94, E95, E96, and L97) (ESI Fig. 4c†). Further, a large decrease in cTnC linker flexibility has been noted upon binding of TnI_1–211_, indicating that conformational dynamics is a fundamental aspect of TnC–TnI interactions during activation of contraction.^37^

The crystal structure of the core domain of the Ca^2+^-saturated cardiac Tn complex revealed a chopstick-like TnI configuration and also some of the TnC–TnI interactions of the open TnC active state.^7^ Some highly flexible Tn segments were not present or were not resolved in the crystal structure, thus indicating that Tn conformational dynamics remains an unexplored avenue for tuning the activation of muscle contraction. The concept of population shifts or re-distribution of conformational states introduces the dependence of protein function on conformational flexibility.^38^ In fact, there are examples of proteins (other than Tn) in which increased flexibility of local segments favors conformational transitions of ensembles.^39^ Our results point out the dynamic behavior of the EF-hand loop in the defunct site I and the D/E linker (Fig. 4i and j). By introducing C84Y and shifting the conformational ensemble toward a more open state, the open active state of WT is mimicked in solution. Therefore, we assessed how cTnC behaves allosterically within the context of the BC helices moving apart from the NAD unit, a sine qua non condition for activation of muscle contraction. Frustration analysis of the cTnC hinge mechanism accommodating the swTnI peptide revealed that most of the interactions within the BC unit are highly frustrated (Fig. 3). Large-scale structural rearrangements such as those occurring in functional hinges have been shown to involve highly frustrated segments in the protein adenylate kinase.^40^ Our data support a model in which cTnC behaves allosterically in the open state prior to muscle activation. This allostery involves a large-amplitude, rigid-body motion of the highly frustrated BC helices driven away from the NAD unit accompanied by local rearrangements of minimally frustrated dynamic segments such as defunct site I and the D/E linker to alleviate the high-stress transition toward the open state.

Our solution experiments and computational simulations allowed us to explore cTnC's sampling of discrete dynamic states and demonstrated that the C84Y variant, due to its location at the end of αD-helix, impairs allosteric communication within cTnC and its conformational dynamics. The concept of minimal frustration, referring to a hypothesis that was developed to quantify the dominance of interactions that stabilize specific native structures over other interactions that favor topologically distinct, non-native structures that serve as energy traps,^41^ is readily applicable to these observations. It helps us to understand how a bulky amino acid within a minimally frustrated region of cTnC could negatively alter the balance of microstates between closed and open conformations by introducing nonnative interactions. Nevertheless, studies of the whole thin filament using cryo-EM are necessary to understand how transitions between the discrete dynamic states in the TnC that we revealed are related to the large-scale conformational changes in within the whole thin filament upon activation by Ca^2+^.

Conflicting molecular contacts could explain why some mutations lead to adverse outcomes and help to clarify some higher-level characteristics of cardiomyopathy observed in our *Tnnc1*^WT/C84Y^ mouse model. In particular, the observation of faster dynamics in regions of cTnC-C84Y in solution, combined with frustration analysis (Fig. 4 and ESI Fig. 6 and 7†), provide significant molecular insights into the altered (faster) dynamics at higher levels of structural organization—specifically, thin filament regulatory units that contain cTnC—inferred from both altered Ca^2+^ sensitivity of isometric force combined with analysis of the force-*k*_TR_ data (Fig. 6d and ESI Table 3†). The force-*k*_TR_ relation (Fig. 6d) informs about both cross-bridge cycling kinetics (ESI Table 3†) and dynamics of individual regulatory units.^42^ Comparing force-*k*_TR_ relations between our *Tnnc1*^WT/C84Y^ (Fig. 6d) with *Tnnc1*^A8V/A8V^ mouse models,^43^ we note that HCM variants in *Tnnc1* do not necessarily cause exactly the same alterations in Ca^2+^-activation kinetics. The low-force end (left end) of the force-*k*_TR_ relation, in particular, is indicative of dynamics of individual regulatory units,^42^ with slower dynamics leading to an elevation of the force-*k*_TR_ relation at low force, and faster dynamics leading to lowering of the force-*k*_TR_ relation at low force^42,44^ as observed here for *Tnnc1*^*WT/C84Y*^ compared with *Tnnc1*^*WT/WT*^ despite no significant change in *F*_max_ (Fig. 6d). This in turn is responsible for faster dynamics of the whole heart observed *in vivo* in *Tnnc1*^WT/C84Y^ mice (ESI Table 1†).

Investigation of cTnC-C84Y therefore provides a platform to visualize how cTnC responds allosterically to Ca^2+^ binding and its molecular partners, and how this leads to alterations at the tissue and organ systems levels. Our physiological measurements confirm early pathological changes in the hearts of *Tnnc1*^WT/C84Y^ mice, which showed early indications of diastolic dysfunction and pathological ventricular hypertrophy. In aged *Tnnc1*^WT/C84Y^ mice, left ventricular hypertrophy and myocardial fibrosis were visibly evident. Our previous studies have shown that cTnC variant residues located in the C-terminus also impact D/E linker dynamics and Ca^2+^ sensitivity of contraction.^3^ Furthermore, the C-terminus of cTnT allosterically modulates contraction through binding to cTnC.^45^ Mutations influencing frustration networks can affect allosteric events either directly or indirectly and may be a unifying mechanism dictating changes in Ca^2+^ affinity of site II that cause HCM.^3,43^ In summary, our findings demonstrate the importance of studying cardiomyopathic mutations at multiple scales of biological complexity and provide a framework for identifying novel therapeutic targets aimed at directly modulating the sarcomere in disease.

## Methods

### Recombinant protein preparation

The pET-3d vectors containing the human sequence of WT or C84Y cardiac TnC were heat shocked into BL21 DE3 strain and prepared as previously described.^3^ Briefly, recombinant, overexpressed protein was obtained by lysis of *E. coli* in 50 mM Tris–Cl (pH 7.8), 6 M urea, 1 mM EDTA, and 1 mM of fresh dithiothreitol (DTT). Clarified supernatants were loaded onto a Q-sepharose column and eluted by a linear gradient from 0 to 600 mM KCl. Fractions were dialyzed overnight against 50 mM Tris–Cl (pH 7.5), 50 mM NaCl, 1 mM MgCl_2_, 1 mM CaCl_2_, and 1 mM DTT, and loaded onto a Superdex 75 preparation grade column to achieve purity higher than 95%. ^15^N- and ^15^N–^13^C-labeled C84Y was produced as described.^3^

### Fluorescence spectroscopy

Fluorescence emission spectra of 2 μM bis-ANS upon excitation at 360 nm were recorded at 440–580 nm and at 15 °C. Purified cTnC WT and C84Y proteins were mixed in 200 mM MOPS (pH 7), 150 mM KCl, 2 mM EGTA, and 1 mM DTT to reach 2 μM final concentration. The *p*Ca 8 and 4 values were calculated using a *p*Ca calculator program^46^ considering 2 mM free Mg^2+^.

### NMR spectroscopy and backbone assignment

We acquired a set of 3D-experiments composed of HNCaCb, CbCa(CO)NH, HN(Ca)CO, and HNCO using ^15^N–^13^C-labeled C84Y cTnC at 25 °C in a Bruker 700 MHz spectrometer equipped with a triple resonance cryogenic probe. The composition of the buffer used was 20 mM MOPS (pH 7.0), 6 mM CaCl_2_, 1 mM MgCl_2_, 100 mM KCl, and 10 mM DTT containing 10% D_2_O. Peak assignments in the cTnC C84Y ^1^H–^15^N HSQC spectrum was guided by superposition of previous WT peak assignments. 3D-experiments were used to unambiguously confirm peak correlations not clearly distinguished by WT *vs.* C84Y spectra superposition. The CCPN suite was used for backbone peak assignments.

### Circular dichroism (CD)

Thermostability assessment of 0.1 mg ml^−1^ WT or C84Y cTnC was carried out in a Chirascan spectropolarimeter. Thermograms were acquired at 222 nm from 20 to 90 °C at a rate of 1 °C min^−1^ every 0.2 °C in a 1 mm quartz cell. Buffer composition was 20 mM MOPS (pH 7.0), 100 mM KCl, and 1 mM EGTA for the Ca^2+^-free (apo) state, and addition of 2 mM MgCl_2_ and 1 mM CaCl_2_ to yield free Ca^2+^ and Mg^2+^ concentration of 0.1 mM and 2 mM, respectively, for the Ca^2+^-bound (primed) state. Mean residue ellipticity values in deg. cm^2^ dmol^−1^ res^−1^ were calculated from [*θ*]_MRE_ = *θ ×* 0.1(MW/*n*)/l × *c* where *θ* is ellipticity in millidegrees, MW is the molecular weight in Daltons, *n* is the number of peptide bonds, *l* is the path length in cm, and *c* is the concentration in mg ml^−1^.

### Small angle X-ray scattering (SAXs) acquisition and analysis

SAXS data were collected on the SAXS1 beam line of the National Synchrotron Light Laboratory (Campinas, Brazil) as previously described.^3^ Parasitic scattering and buffer contribution were properly subtracted for each condition. Chemical-induced unfolding of 2 to 4 mg ml^−1^ cardiac TnC was performed with urea concentrations of 1, 3, 5, and 7 M. The buffer composition was 200 mM MOPS (pH 7.0), 100 mM KCl, and 1 mM EGTA for the Ca^2+^-free (apo) state and in addition of 2 mM MgCl_2_ and 1 mM CaCl_2_ to yield free Ca^2+^ and Mg^2+^ concentrations of 0.1 mM and 2 mM, respectively, for the Ca^2+^-bound (primed) state. Kratky plots were obtained by multiplying the scattering intensity *I*(*s*) by the scattering vector, *s* squared and the results were plotted as a function of *s*.^47^

### Temperature analysis by NMR spectroscopy

We acquired ^1^H–^15^N heteronuclear single quantum correlation (HSQC) spectra of WT and C84Y cTnC at increasing temperatures ranging from 15 to 50 °C in a Bruker 700 MHz spectrometer. ^1^H–^15^N HSQC spectroscopy generates 2D spectra with one axis for protons (^1^H) in amide hydrogens and the other for a heteronucleus, ^15^N in this case, and the spectra contain one peak for each unique proton attached to each heteronucleus. This provides a unique fingerprint for each protein and allows detection of residues perturbed by temperature that vary between the C84Y variant compared to WT. The temperature dependence of ^1^H chemical shift (*δ*) for each ^1^H–^15^N peak correlation was fitted using linear regression, and temperature coefficients were obtained from the slope in ppb °C^−1^. Because of the linear dependence of *δ* to temperature increments, NMR assignment of ^1^H–^15^N HSQC spectra at increasing temperatures was done by tracking *δ* at 25 °C as a reference.

### TnI peptide titrations by NMR spectroscopy

Switch (sw) and inhibitory (i) TnI peptides were synthesized by Genscript (Piscataway, Inc.) with purity higher than 95% and contained acetylation and amidation at the N- and C-terminal ends, respectively. A shorter switch TnI peptide (swTnI_147–163_) precipitated at concentrations above ∼50 μM and thus data from higher concentrations were excluded from analysis. To circumvent this, we performed most experiments with a longer and more soluble switch TnI peptide (swTnI_144–165_).

We acquired ^1^H–^15^N HSQC spectra at 37 °C of WT and C84Y cTnC (primed state) in the absence of peptides, and in the presence of increasing concentrations of each peptide (15, 40, 70, 120, 150, and 200 μM). Chemical shift analysis (CSA) comparing WT against C84Y or WT/C84Y peptide-bound against peptide-free cTnC was performed by the following equation: CSP = [(Δ*δ*_H_)^2^ + 0.1(Δ*δ*_N_)^2^]^0.5^ where Δ*δ*_H_ and Δ*δ*_N_ are the chemical shift variations of ^1^H and ^15^N, respectively, for the two conditions studied.

### Dynamic properties

Longitudinal (*R*_1_) and transverse (*R*_2_) relaxation rates of C84Y were acquired from ^1^H–^15^N HSQC spectra with relaxation delays of 20, 50, 100, 200, 250, 500, 750, 1000, and 1500 ms for *R*_1_ and 16, 48, 80, 112, 144, 176, 208, 240, 272, and 304 ms for *R*_2_. Carr-Purcell-Meiboom-Gill (CPMG) relaxation dispersion experiments were acquired in a Bruker 700 MHz spectrometer at 25, 32, and 37 °C. Effective *R*_2_ relaxation rates (*R*_2eff_) as a function of residues were obtained from ^1^H–^15^N HSQC spectra acquired at CPMG frequencies of 50 and 1000 MHz to assess intermediate exchange dynamics of C84Y. *R*_2eff_ was calculated from the following equation *R*_2eff_ = −1/*T* ln(*I*_0_/*I*_CPMG_) where *T* is the relaxation time and *I*_0_ and *I*_CPMG_ are the intensities of resonances obtained from experiments in the absence or presence of the CPMG pulse block, respectively.

### Molecular dynamics (MD) simulations

#### TnC–TnI peptide

MD simulations were performed with GROMACS 2018.^48^ Starting structures were the best representative conformer from the 1AP4 (ref. 49) (WT TnC) and 1MXL^8^ (WT TnC bound to the swTnI_147–162_ peptide) ensembles, models 14 and 18, respectively. Position 84 was changed to Tyr for the C84Y trajectories using Chimera,^50^ and selecting the most probable rotamer conformation. The CHARMM36m force field was used with the CHARMM TIP3P water model.^51^ Na^+^ and Cl^−^ ions were added to achieve charge neutrality and a physiological salt concentration of 0.15 M. System energy was minimized by steepest descent and propagated by leapfrog integration with a 2 fs time step. Temperature and pressure were regulated at 310 K and 1 bar using *v*-rescale and Parrinello-Rahman algorithms, respectively.^52^ Six replicates of each system were simulated with different initial atom velocity distributions for 3 μs apiece, giving 18 μs total sampling per system, and an aggregate simulation time of 72 μs. Analysis was performed on the concatenated trajectories (6 × 3 μs) for each system. AB interhelical angles were calculated using VGM^53,54^ using residues 14–25 and 38–47 for helices A and B, respectively. Open states were defined by an AB interhelical angle < 110°.^16^ Principal component analysis (PCA) was performed as described previously^55^ using the C_α_ atoms of the cTnC core region (residues 4–85).

#### Troponin complex and thin filament

MD simulations of the troponin (Tn) complex were performed based on the crystal structure resolved at 3.3 Å resolution by Takeda *et al.*^7^ and deposited in the Protein Databank (PDB ID 1J1E). We followed the procedure outlined in ref. 56 to add residues in TnI (residues 1–34, 137–146 and 192–210) and TnT (residues 280–288) that were not resolved in the Takeda *et al.* structure. The modeled structure, hereafter referred to as ‘intact Tn’, thus consisted of TnC 1–161, TnI 1–172, and TnT 236–285. Additionally, we simulated the Tn complex bound to tropomyosin and actin from the thin filament structure resolved *via* electron microscopy. We refer to these structures as the thin filament–troponin (TF–Tn) configuration. We used two structures corresponding to the Ca^2+^-free and Ca^2+^- bound states (PDB 6KN7 and 6KN8 ^9^), which were resolved at 6.6 and 4.8 Å, respectively. Thus we have the TF–Tn Ca^2+^-bound model and TF–Tn Ca^2+^-free model. To maintain a computationally tractable system, we limited the structures to Tn as well as the two adjacent actins and tropomyosin helices. This led to a truncated system with 1330 residues. Given the low resolution of the 6KN7 and 6KN8 structures, the positions of bound Ca^2+^ ions were not resolved. We therefore placed two Ca^2+^ ions in 6KN7 sites III and IV (apo, C-terminal domain) and in 6KN8 sites II, III and IV (holo, N- and C-terminal domains) based on their positions in the Takeda *et al.*^7^ Tn structure. Lastly, we utilized the tyrosine rotamer at position C84Y, based on our simulations of the isolated N-terminal domains (1MXL and 1AP4).

These Tn structures were solvated using TIP3P waters^57^ and assuming a 14 Å margin around the protein for the periodic box. The ionic strength was set to 0.15 M NaCl after adding counter ions to neutralize the charge using the CHARMM36m force field.^51^ The MD protocol was otherwise identical to that used in 1MXL/1AP4 simulations, with exception that triplicate, minimum 100 ns simulations were performed. We performed the RMSF calculations and contact map analyses using the CPPTRAJ program.^58^ A contact was defined with an atom-atom distance cutoff of 7 Å for residue pairs separated by at least 6 residues. In the RMSF calculations, all frames were first aligned to the first frame using the backbone atoms of TnC residues M1- L100. All code written in support of this publication is publicly available at https://github.com/huskeypm/pkh-lab-analyses/(2021-TnSims).

### Spatial localization of frustration nets

We used the Frustratrometer (http://www.frustratometer.tk/)^59^ to localize the level of frustration for each residue interaction. The theoretical calculation generates a frustration index, that measures “how favorable a particular contact is relative to sets of possible interactions, normalized using the variance of the energy distribution”.^60^ The color scale indicates the local mutational frustration where the minimally, neutral and highly frustrated sites are shown in green, gray and red, respectively. PDB models (Fig. 3: 1SPY, 1AP4 and 1MXL); (ESI Fig. 7†: 1TCF, 1J1E).

### Animal procedures

The experimental protocol involving mice was reviewed and approved by the Florida State University (FSU) Animal Care and Use Committee (ACUC). Animal handling and experiments were performed in accordance with the Guide for the Care and Use of Laboratory Animals outlined by the National Institutes of Health (NIH). Animals were housed in a temperature-controlled vivarium on a 12:12 h light/dark cycle at FSU with *ad libitum* access to water and normal chow. For terminal procedures, mice were humanely sacrificed by rapid cervical dislocation under anesthesia with isoflurane, USP (Patterson Veterinary). Ages of the mice ranged from 3–14 months and mixed sexes were used as indicated in each respective figure legend.

### Genetically engineered *Tnnc1*-p.C84Y mouse model

A gene-targeting approach was utilized to alter one *Tnnc1* gene in the mouse genome to express *Tnnc1*-p.C84Y by homologous recombination in murine 129/SvEV-derived embryonic stem (ES) cells. The sequential overlapping polymerase chain reaction (PCR)-based method was used to generate the genetic variant in exon 4 of a genomic clone encompassing the entire murine *Tnnc1* gene. The construct was subcloned into the “Targeting Vector” containing an MC1 neo cassette flanked by two loxP sites and thymidine kinase (TK) under the control of 3′ phosphoglycerate kinase (PGK).

Ella-Cre mice were on an FVB/N strain; *Tnnc1*^WT/C84Y^ and *Tnnc1*^WT/WT^ maintained on a C57BL/6 background.

Mouse genotyping was performed using tail DNA by PCR analysis with the following primers:

*Tnnc1*_WT_Forward: AGTCTGAGGAGGAGCTGTCG.

*Tnnc1*_WT_Reverse: GTCTCACCTCTCATTGGATGC (460 bp fragment).

*Tnnc1*_Neo_Forward: AGGACATAGCGTTGGCTACC (253 bp fragment).

*Tnnc1*_C84Y: CGTGGATTGCTGGAATGC.

### Echocardiography

A Vevo 2100 high-resolution *in vivo* imaging system (Fujifilm Sonosite Inc., Toronto ON, Canada) was used to perform echocardiography (ECHO) analysis as previously described.^61^ Briefly, experimental mice were subjected to light anesthesia with 2% isoflurane (Patterson Veterinary). M-mode imaging of the parasternal short axis view permitted evaluation of wall thickness and left ventricular systolic/diastolic function. Mitral valve flow parameters were collected using pulsed-wave spectral Doppler mode *via* four-chamber viewing of the heart.

### Histopathology

Whole mouse hearts were surgically excised and perfused in 10% buffered formaldehyde solution as previously described.^61^ Fixed hearts were sliced and stained by IDEXX Incorporated (Boston MA, USA). Images were acquired on a Nikon AZ 100M microscope and analysis was performed in a blinded manner using NIH ImageJ software. For Masson's trichrome imaging hearts were stained using Sigma-Aldrich Trichome stain (Masson) kit HT15-1KT according to the manufacturer's directions.

### Cardiac muscle mechanics

Preparation and compositions of the buffered Ca^2+^ solutions and methods for assessment of cardiac muscle mechanical properties were previously described in detail.^43^ Briefly, left ventricle papillary muscle bundles were dissected from freshly excised *Tnnc1*^WT/C84Y^ or *Tnnc1*^WT/WT^ mouse hearts at 6 months of age and permeabilized with 1% Triton X-100 (v/v) in relaxing (*p*Ca 8.0) solution for 4 h at 4 °C. These permeabilized cardiac muscle preparations (CMPs) were stored at −20 °C in 51% glycerol (v/v) in *p*Ca 8.0 and used for experiments within 1 week. Force was measured using a force transducer (Aurora Scientific Inc. model 403A) and length was controlled using a high-speed servomotor (Aurora Scientific Inc. model 322C). Ca^2+^-activated steady-state, isometric tension generation, kinetics of isometric tension redevelopment (*k*_TR_), sinusoidal stiffness (SS) measurements (0.2% *L*_0_ peak-to-peak at 100 Hz) and mathematical modeling of *k*_TR_ were carried out as previously described.^43^ Steady-state data were fitted with either 2-, 3- or 4-parameter Hill equations in SigmaPlot (v.12.0) as indicated in each respective figure legend, whereas estimations of the 3-state model parameters were computed in MatLab as previously described.^43,45^

### Statistics and reproducibility

Ca^2+^ titrations by fluorescence spectroscopy and thermostability measurements by CD are expressed as mean ± S.E.M. Sample size was set to *n* = 3 of at least two protein batches.

SAXS analysis was performed by taking six frames of 5 s each and 2 s delay per frame. Frames were superimposed to inspect for radiation damage. Because successive frames revealed no radiation effects, the six replicates were averaged to enhance the signal-to-noise ratio. Scattering in each urea condition was performed twice (sample size, *n* = 2) from two independent protein batches. Data shown are from one of the samples.

Residues selected in chemical shift perturbation analysis (CSP) from NMR data refer to those that deviate from the average values of CSP (Δ*δ*) among residues + 1 S.D. (Δ*δ* + 1*σ*) and + 2 S.D. (Δ*δ* + 2*σ*).

Histopathology images, echocardiography data and contractile parameters were obtained from both female and male *Tnnc1*^WT/WT^ and *Tnnc1*^WT/C84Y^ mice. Data are presented as mean ± S.E.M. Statistical significance was tested using Student's *t*-test in which alpha *p* < 0.05 (ECHO) and *p* < 0.02 (contractile parameters) were considered to be significant.

## Data availability statement

All data and biological materials generated in this study are available from the corresponding authors upon request. The source data underlying Fig. 2b, 4g–h, 5, 6 and Supplementary Fig. 2a–b, 3a–s, 4b–c, 4h–i, 11b–i are provided in an excel file as a supplementary material.

## Author contributions

M. A. M., G. A. P. d. O., and J. R. P. conceptualization; M. A. M., M. L.-V., A. H. M., B. S., K. M. D. J., E. C., I. C. V., P. M. K.-H., and G. A. P. d. O. data curation; M. A. M., M. L.-V., A. H. M., B. S., K. M. D. J., E. C., M. S. P., I. C. V., P. B. C., P. M. K.-H., and G. A. P. d. O. formal analysis; M. A. M., G. A. P. d. O., and J. R. P. supervision; M. A. M., M. L.-V., A. H. M., B. S., K. M. D. J., E. C., M. S. P., I. C. V., V. E. G., P. B. C., P. M. K.-H., G. A. P. d. O., and J. R. P. investigation; M. A. M., M. L.-V., A. H. M., B. S., J. R. J., K. M. D. J., E. C., M. S. P., I. C. V., P. B. C., P. M. K.-H., G. A. P. d. O., and J. R. P. methodology; M. A. M., and G. A. P. d. O. writing-original draft; M. A. M., G. A. P. d. O., and J. R. P. project administration; M. A. M., M. L.-V., A. H. M., B. S., J. R. J., K. M. D. J., E. C., M. S. P., J. L. S., V. E. G., P. B. C., P. M. K.-H., G. A. P. d. O., and J. R. P. writing-review and editing; E. A. C., J. L. S., P. M. K.-H., G. A. P. d. O., and J. R. P. resources; J. L. S., P. M. K.-H., G. A. P. d. O., and J. R. P. funding acquisition.

## Conflicts of interest

There are no conflicts to declare.

## Supplementary Material

SC-012-D1SC01886H-s001

SC-012-D1SC01886H-s002

SC-012-D1SC01886H-s003

SC-012-D1SC01886H-s004

SC-012-D1SC01886H-s005

SC-012-D1SC01886H-s006

SC-012-D1SC01886H-s007

SC-012-D1SC01886H-s008

SC-012-D1SC01886H-s009

SC-012-D1SC01886H-s010

SC-012-D1SC01886H-s011

SC-012-D1SC01886H-s012

SC-012-D1SC01886H-s013

SC-012-D1SC01886H-s014

SC-012-D1SC01886H-s015

SC-012-D1SC01886H-s016

SC-012-D1SC01886H-s017
