# Supplementary figures and images for: Anomalous structural dynamics of minimally frustrated residues in cardiac troponin C triggers hypertrophic cardiomyopathy
Source: Chem Sci. 2021 Apr 29;12(21):7308–23. doi: 10.1039/d1sc01886h (PMC8171346; doi:10.1039/d1sc01886h)

iTnl<sub>128-147</sub>

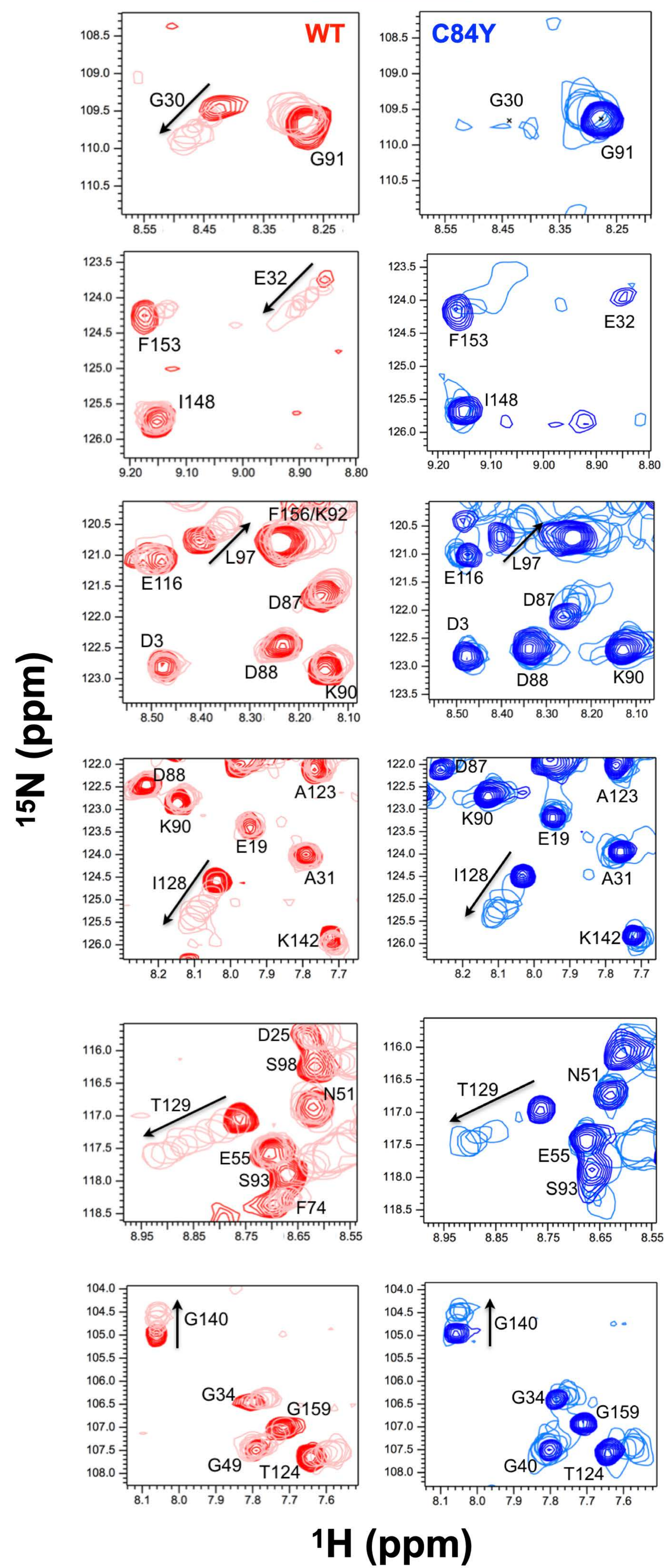

Supplement: SC-012-D1SC01886H-s002 [file SC-012-D1SC01886H-s002.pdf]

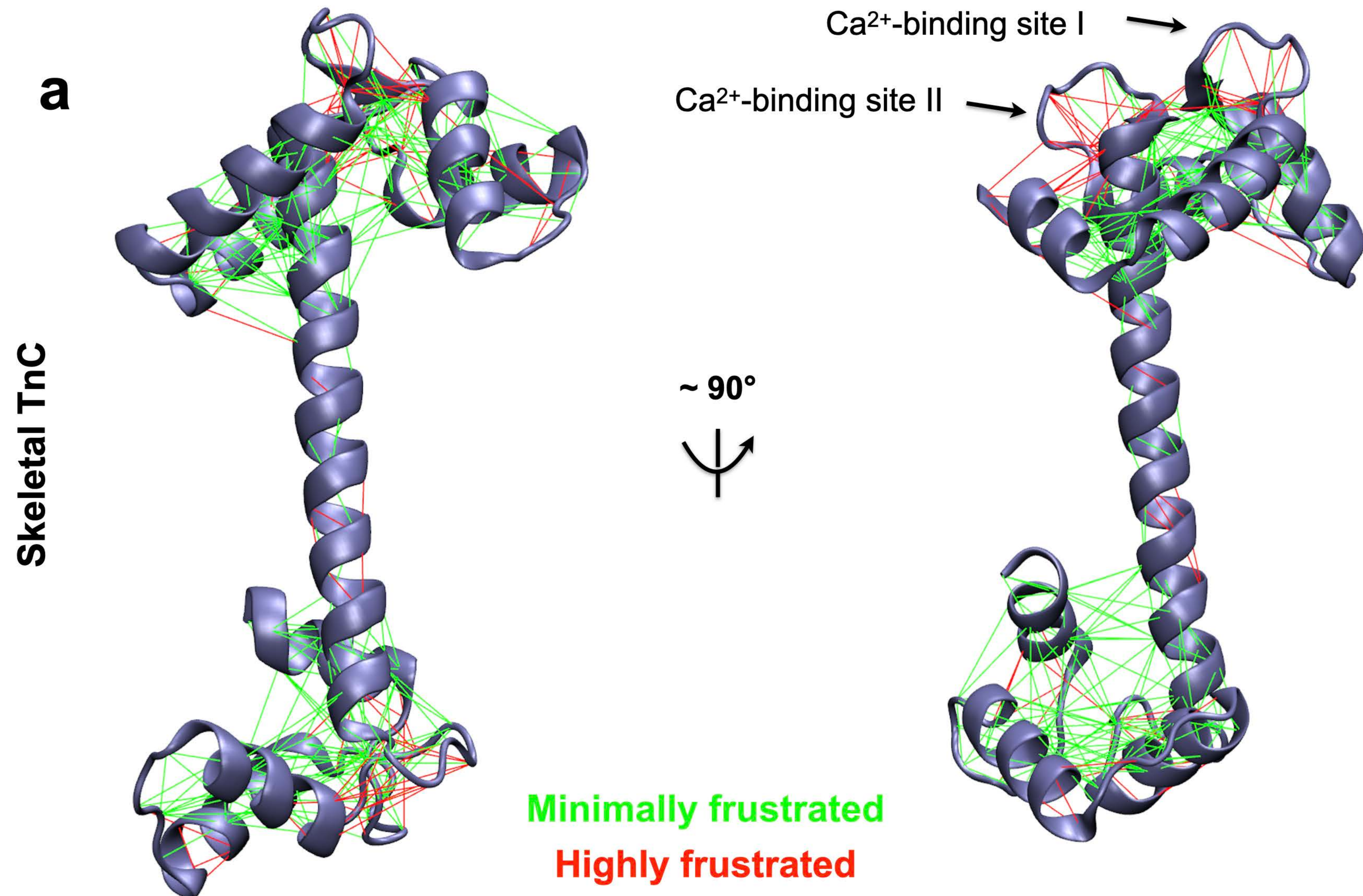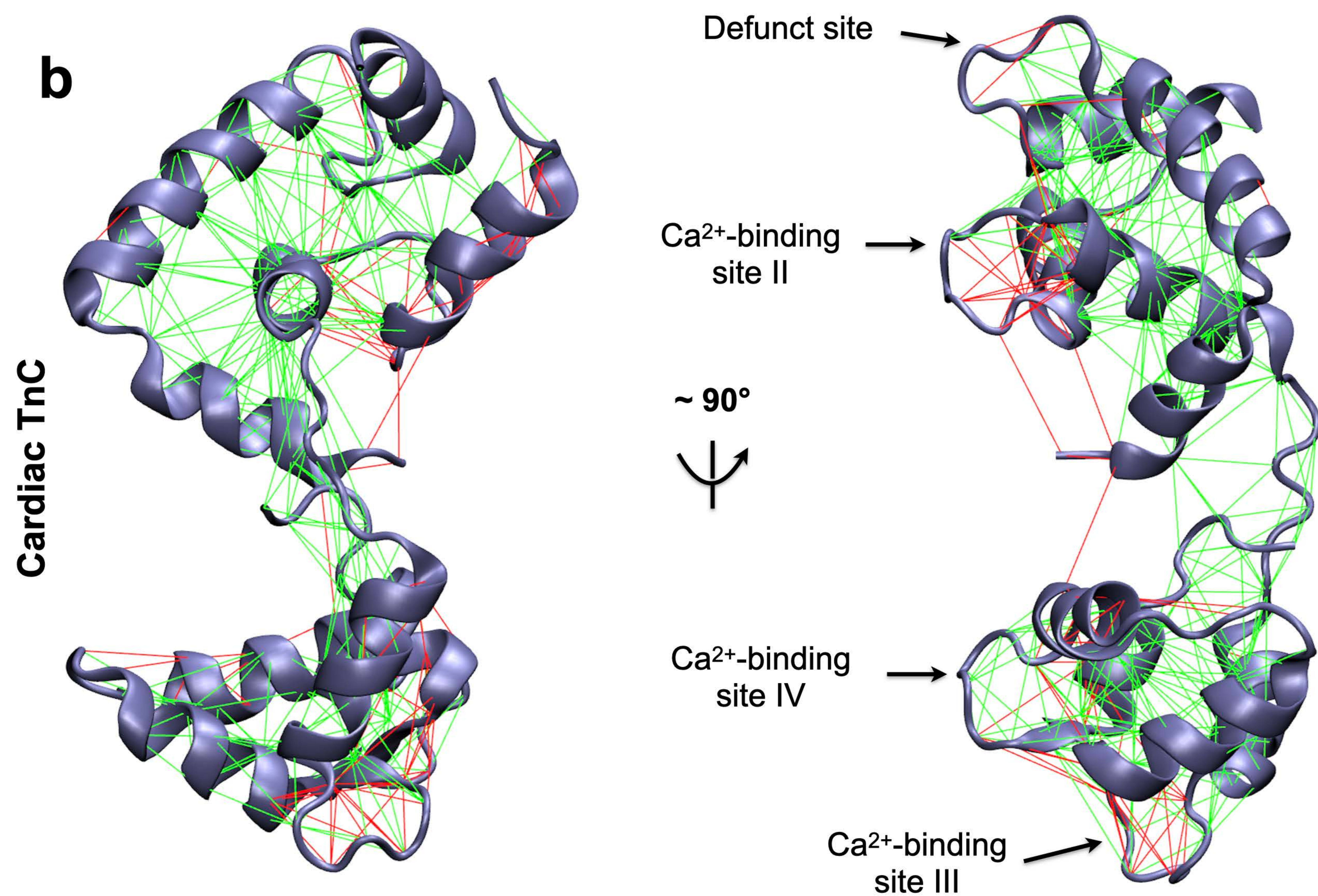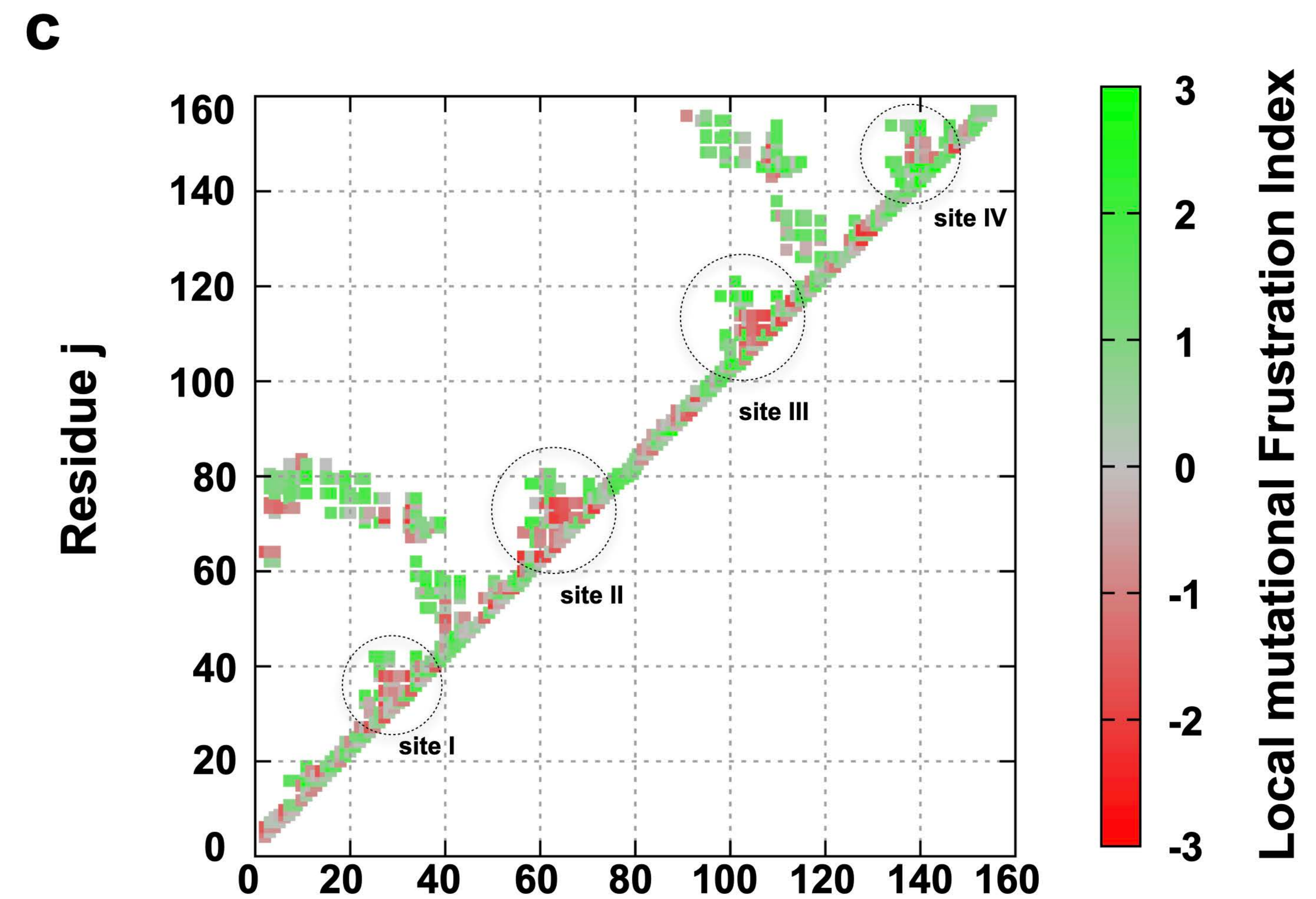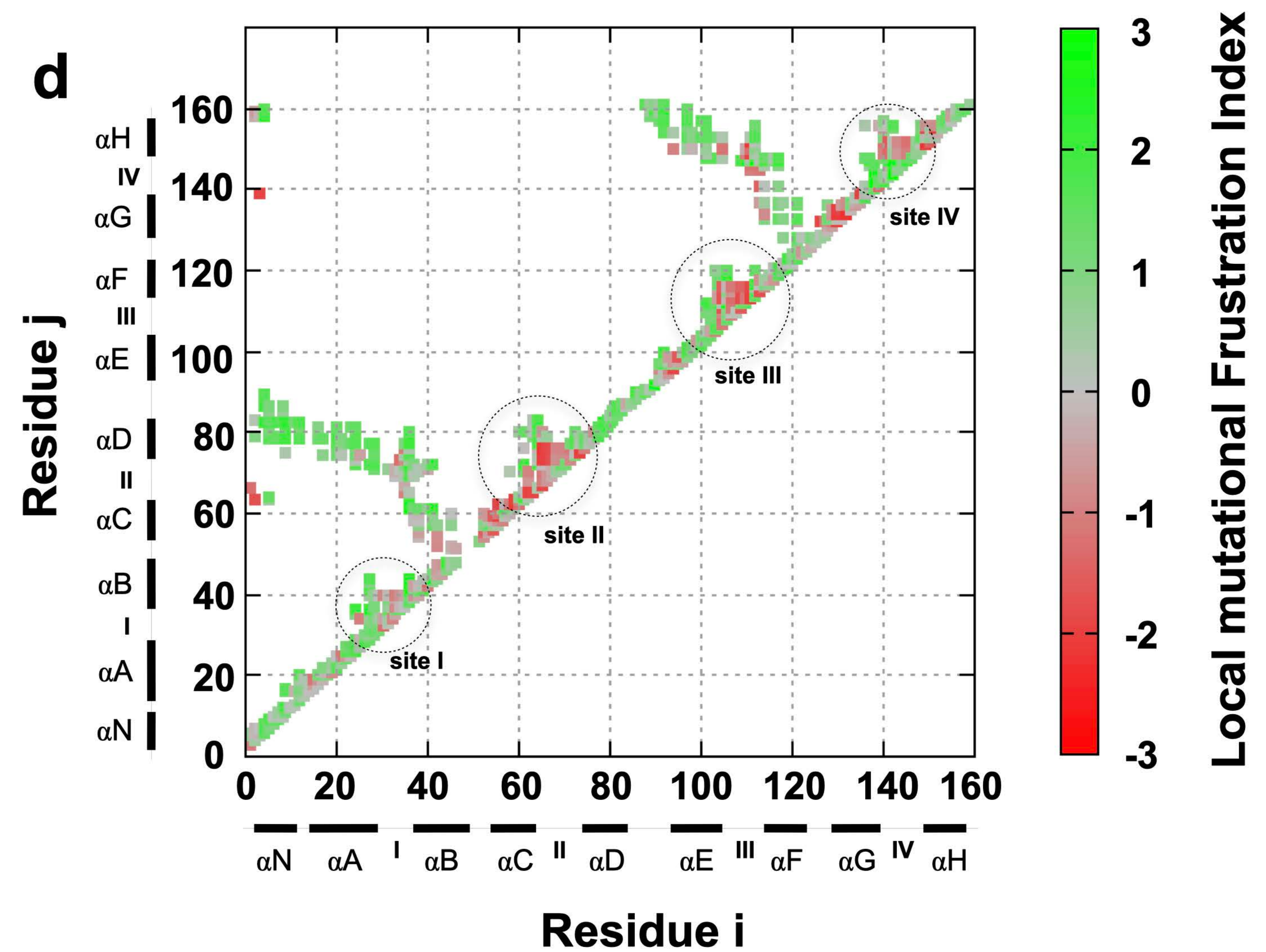

Supplement: SC-012-D1SC01886H-s003 [file SC-012-D1SC01886H-s003.pdf]

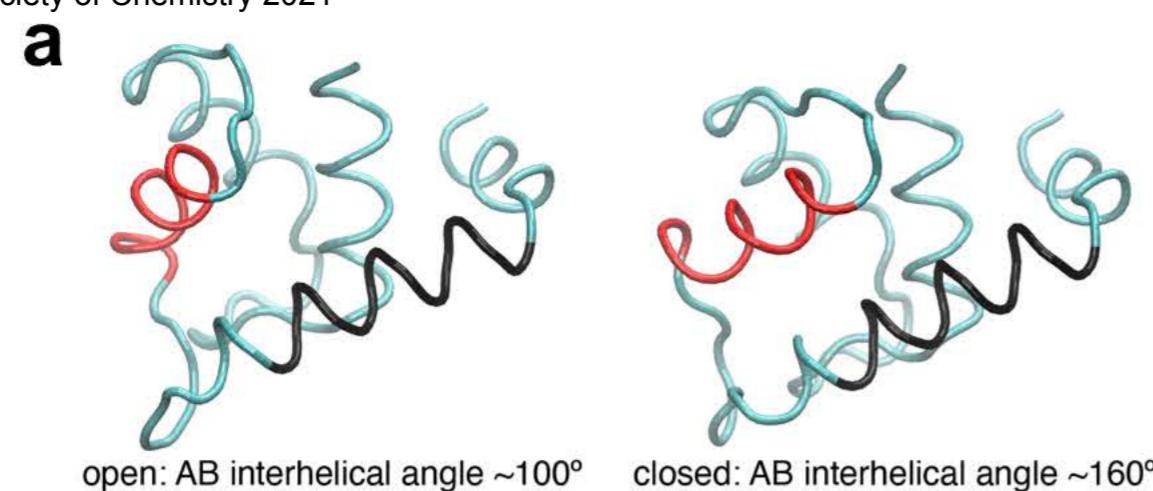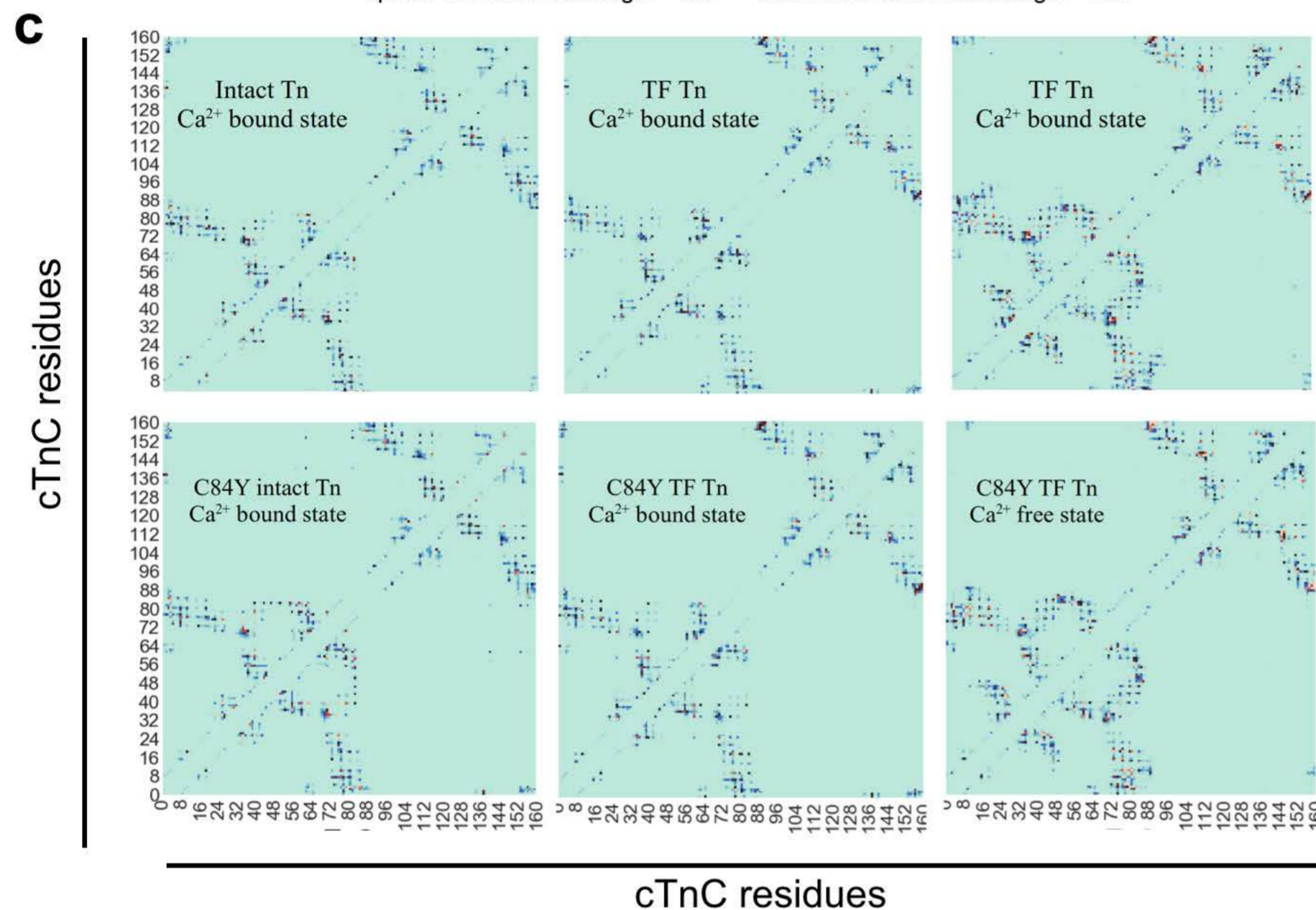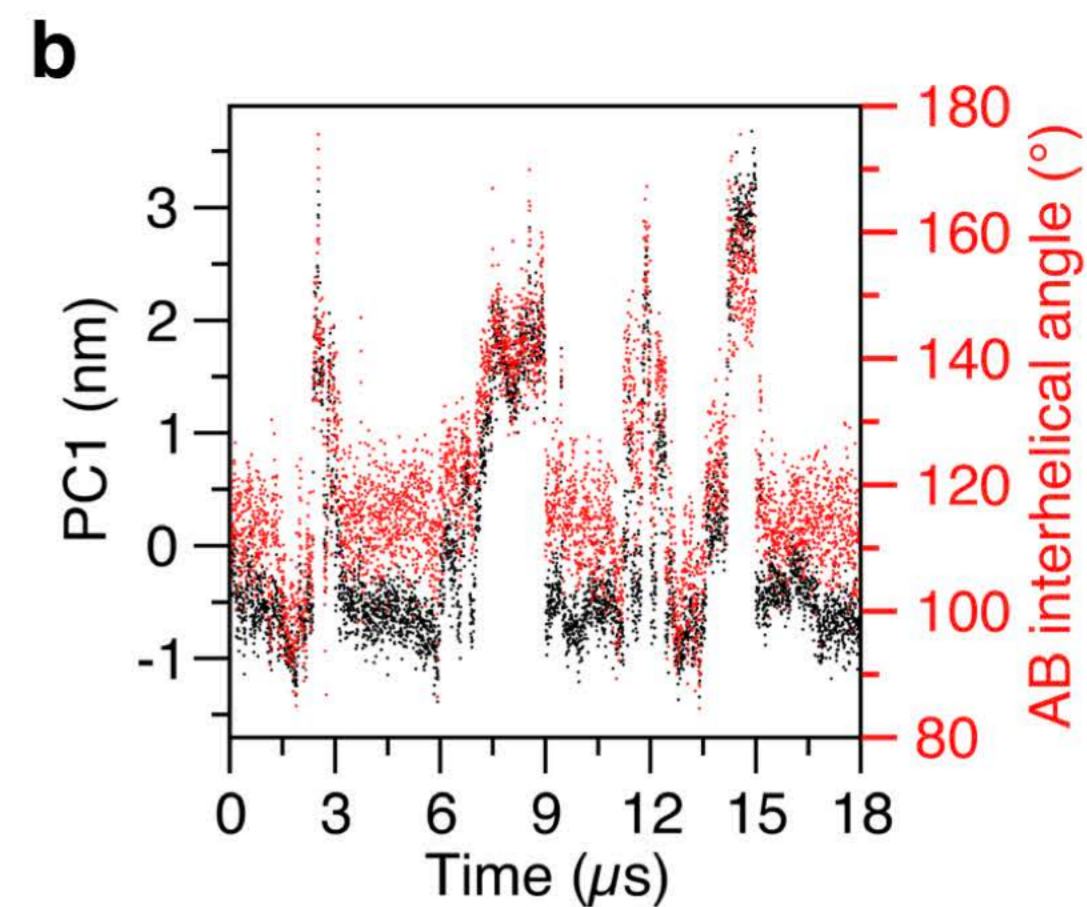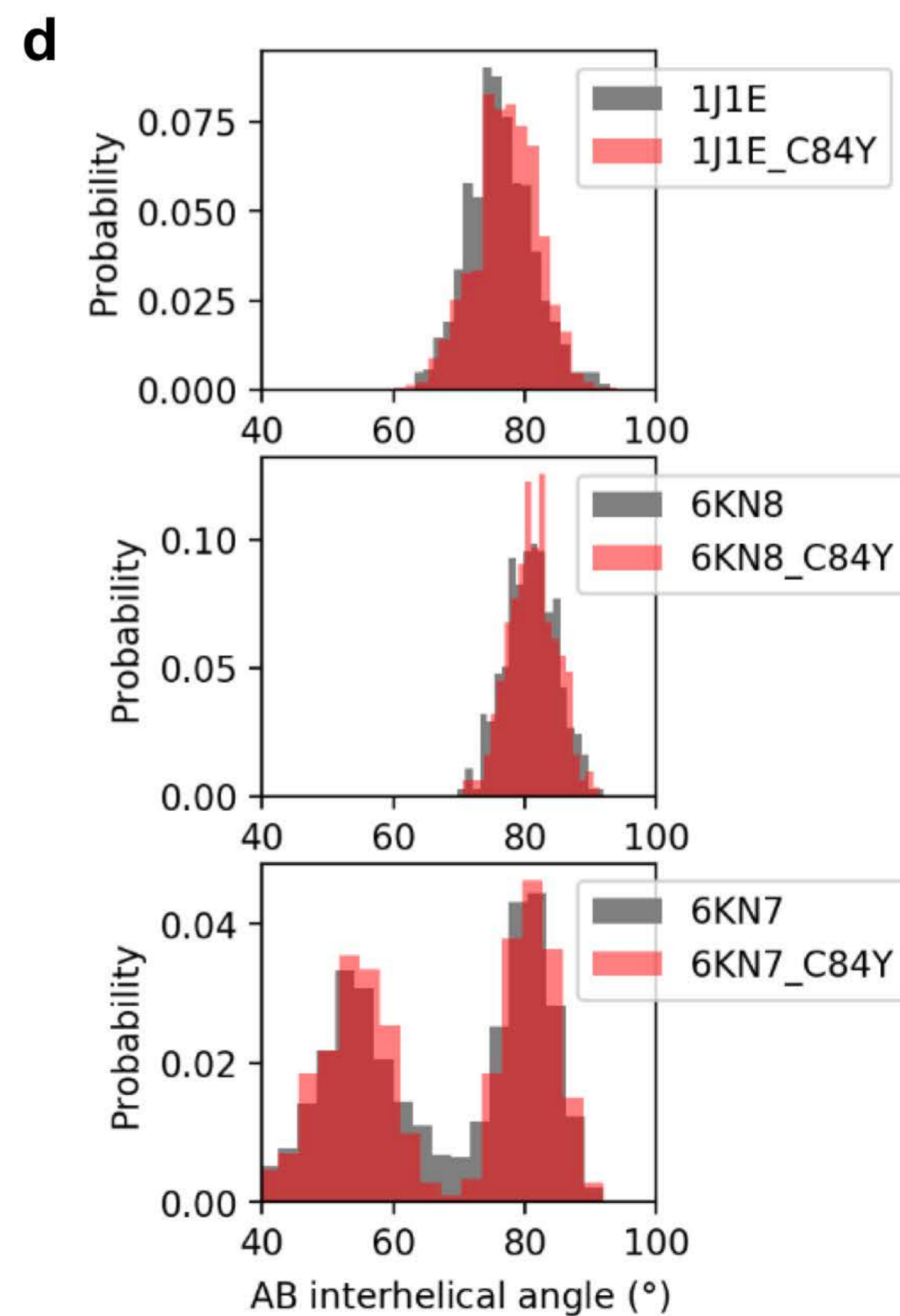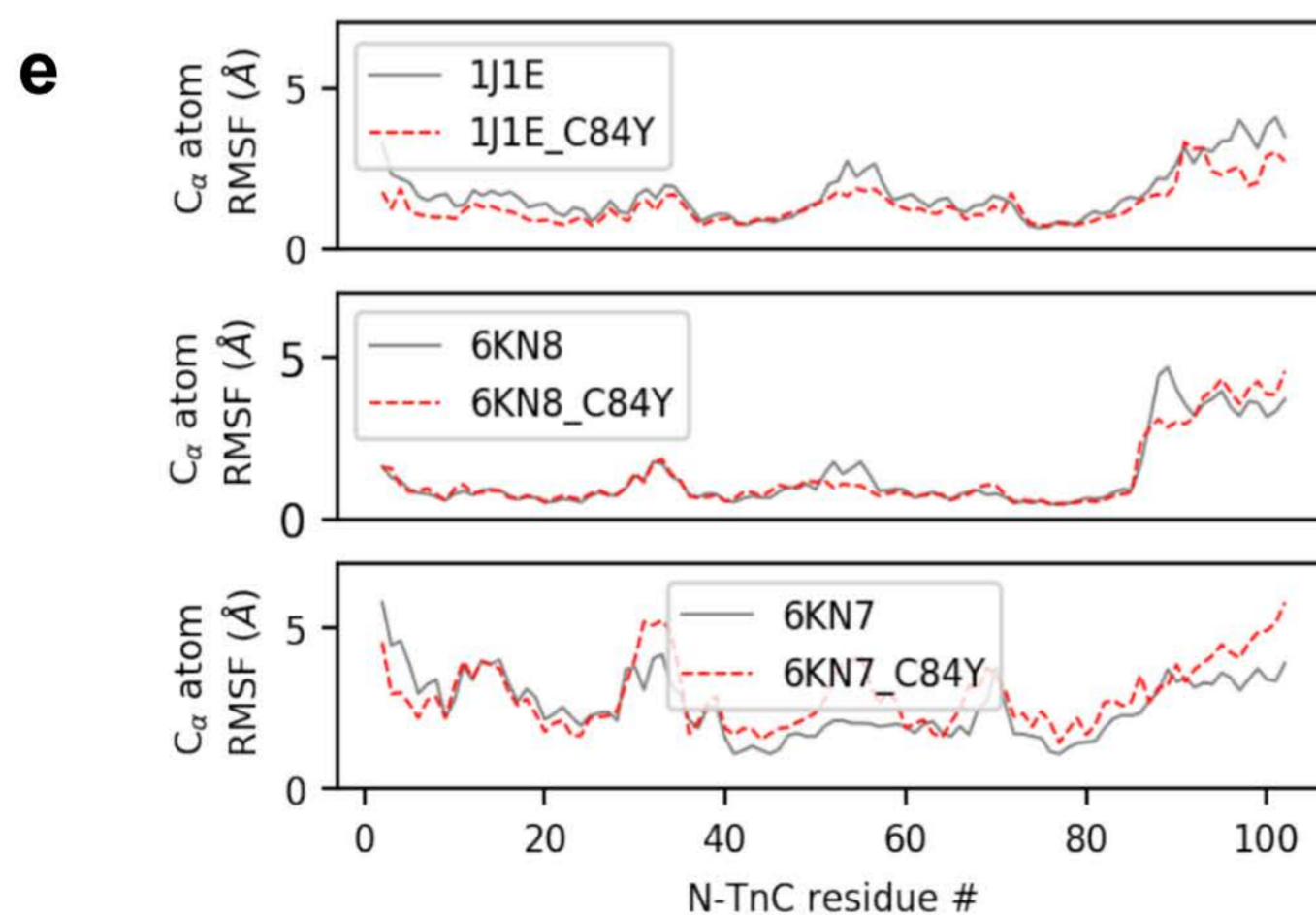

Supplement: SC-012-D1SC01886H-s004 [file SC-012-D1SC01886H-s004.pdf]

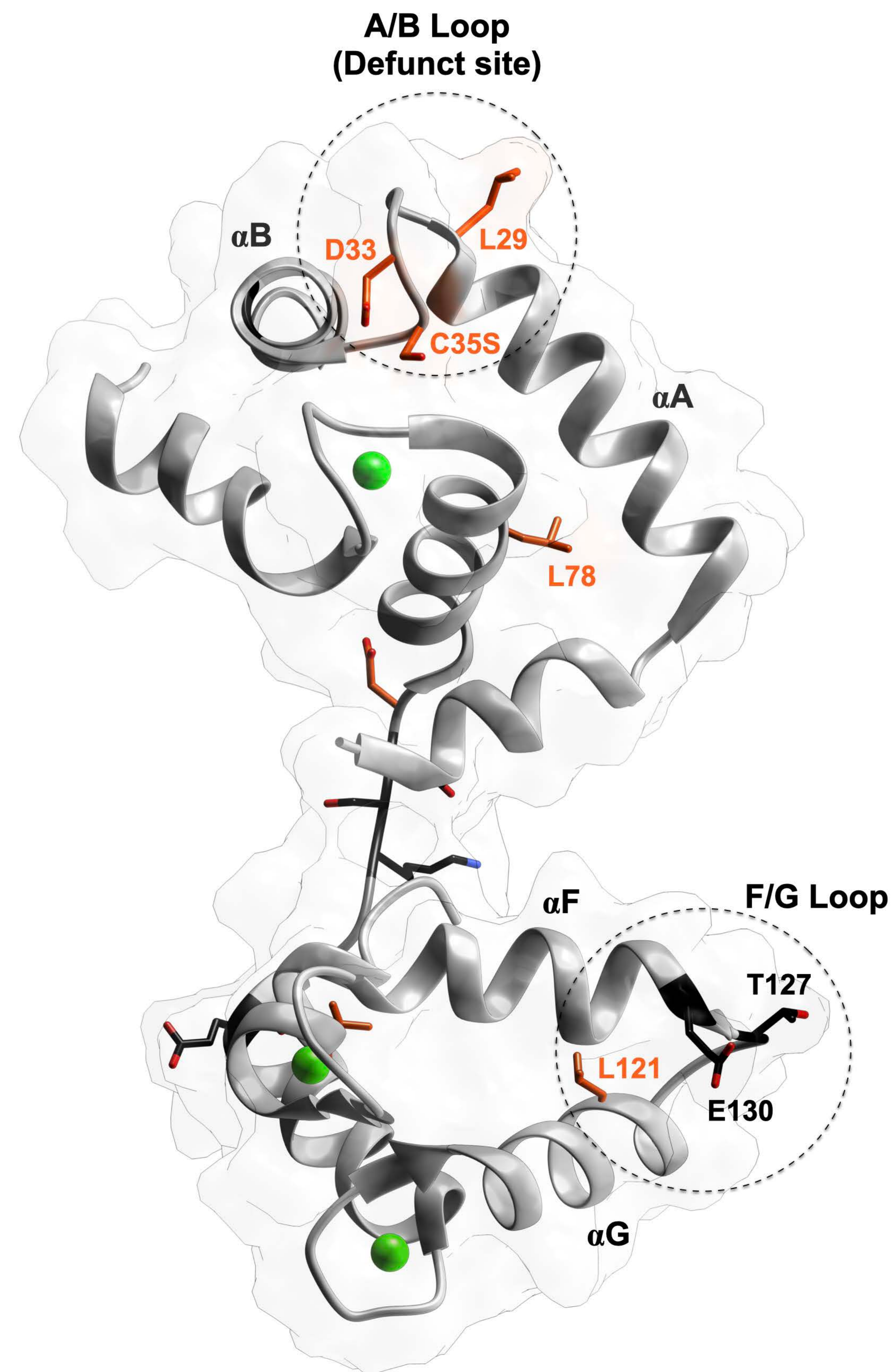

$\sim 90^\circ$

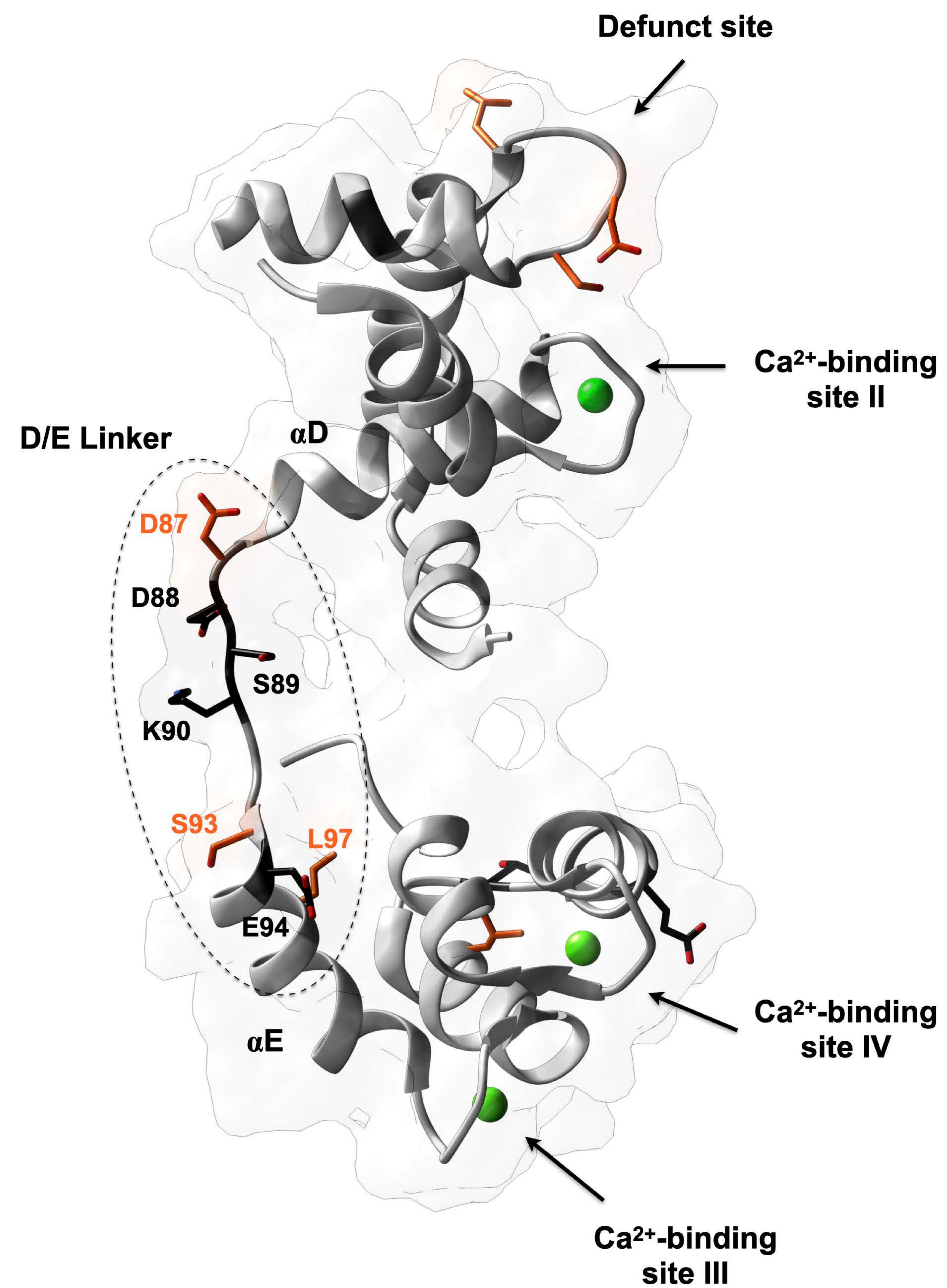

Supplement: SC-012-D1SC01886H-s005 [file SC-012-D1SC01886H-s005.pdf]

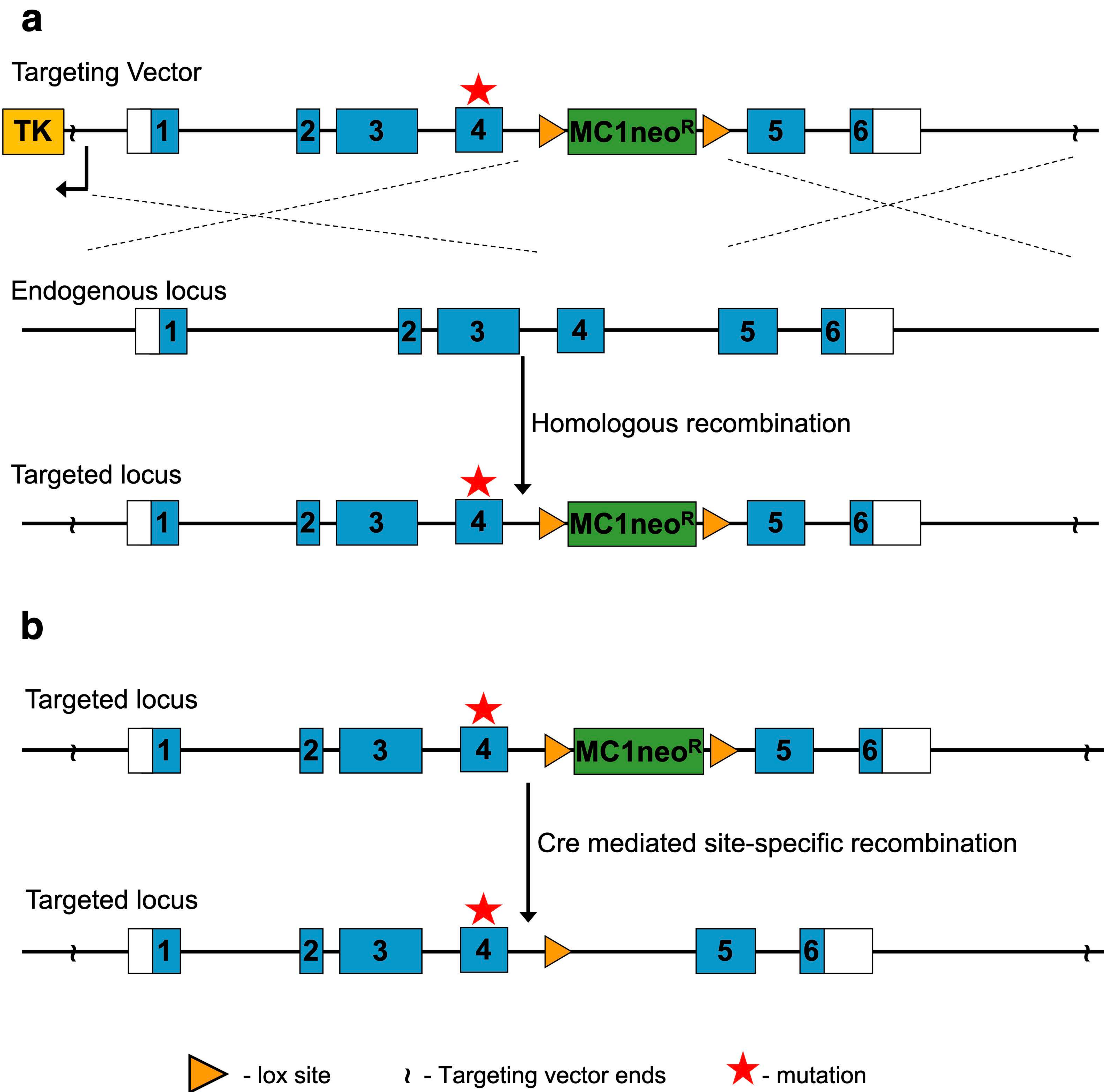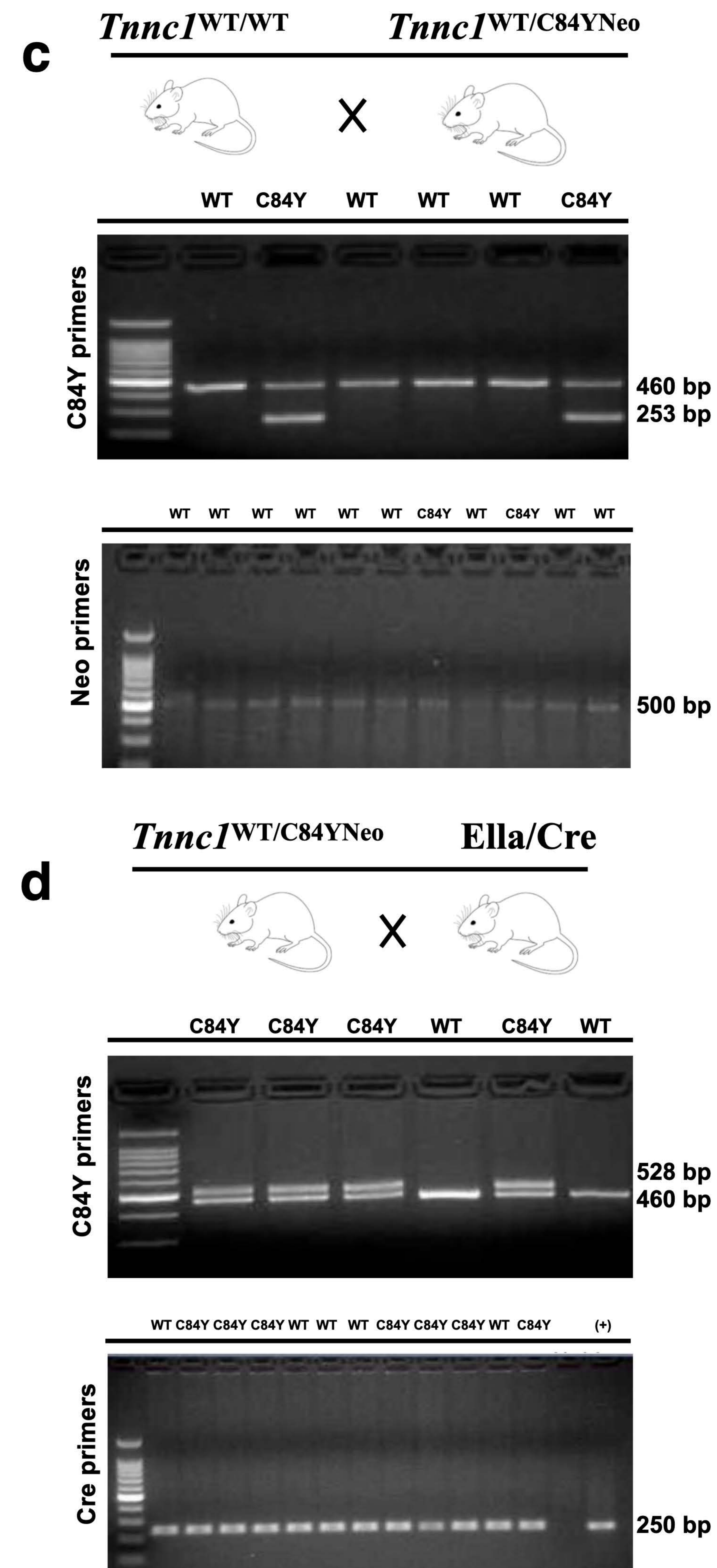

Supplement: SC-012-D1SC01886H-s006 [file SC-012-D1SC01886H-s006.pdf]

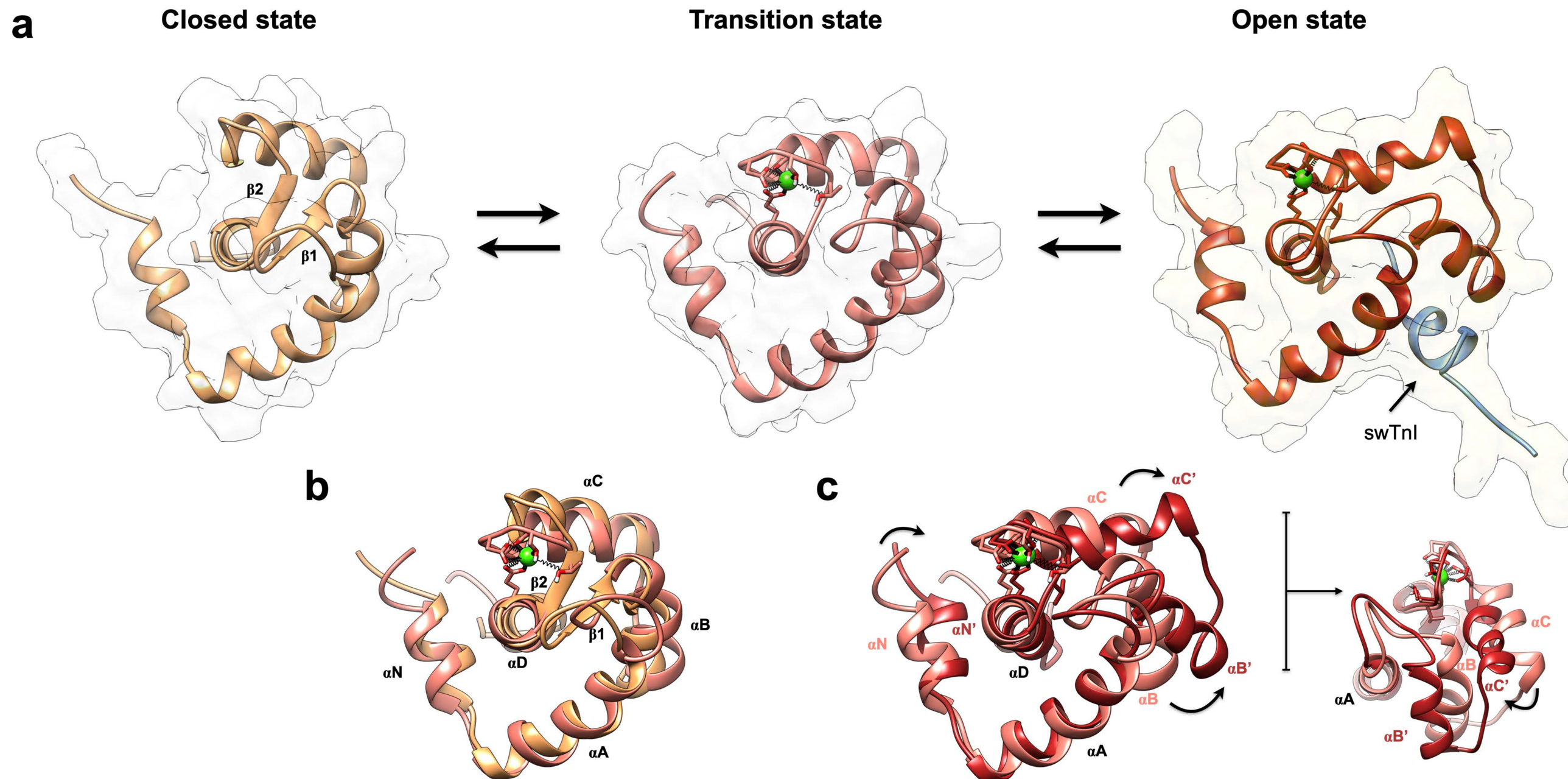

Supplement: SC-012-D1SC01886H-s012 [file SC-012-D1SC01886H-s012.pdf]

**a**

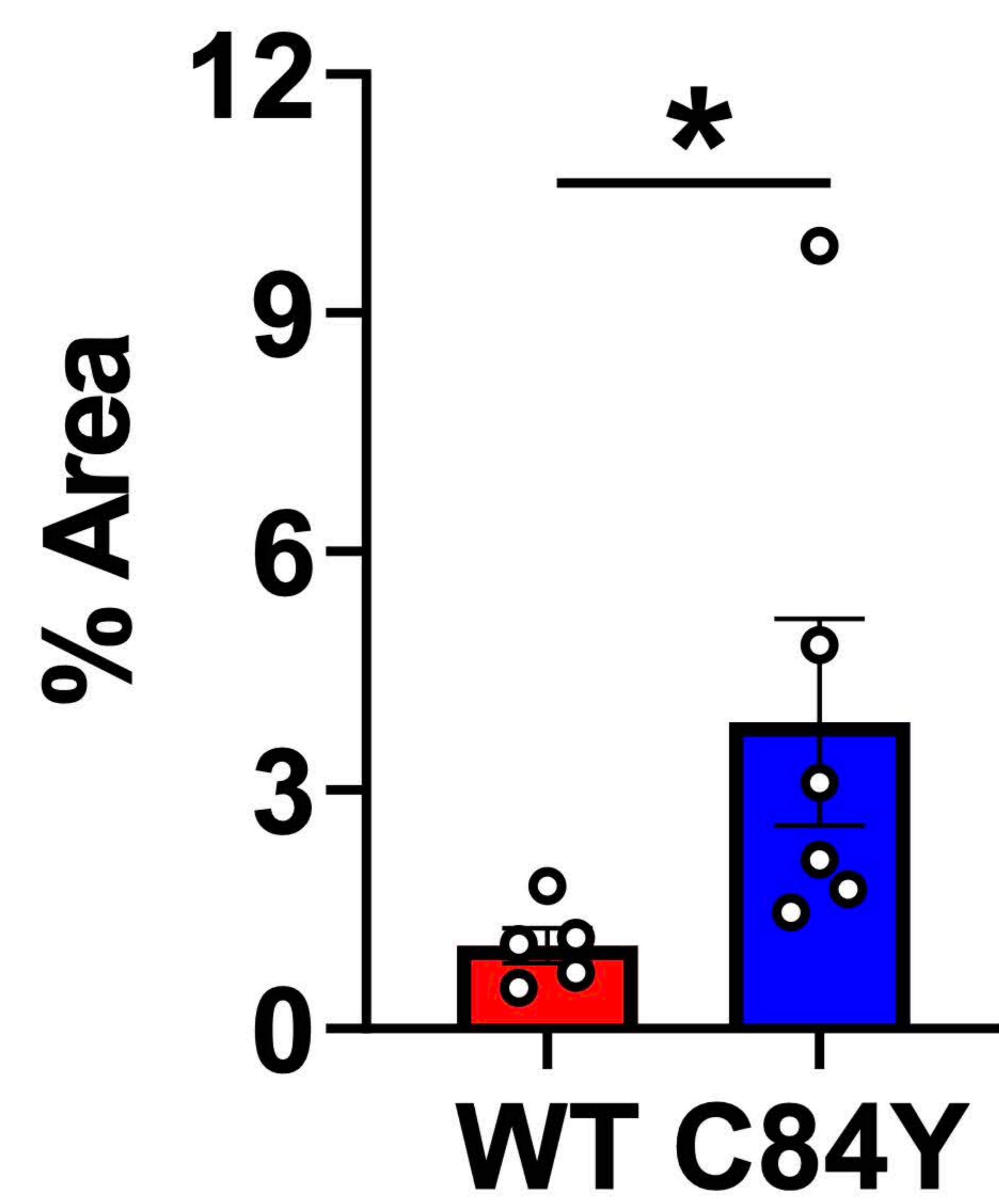

**b**

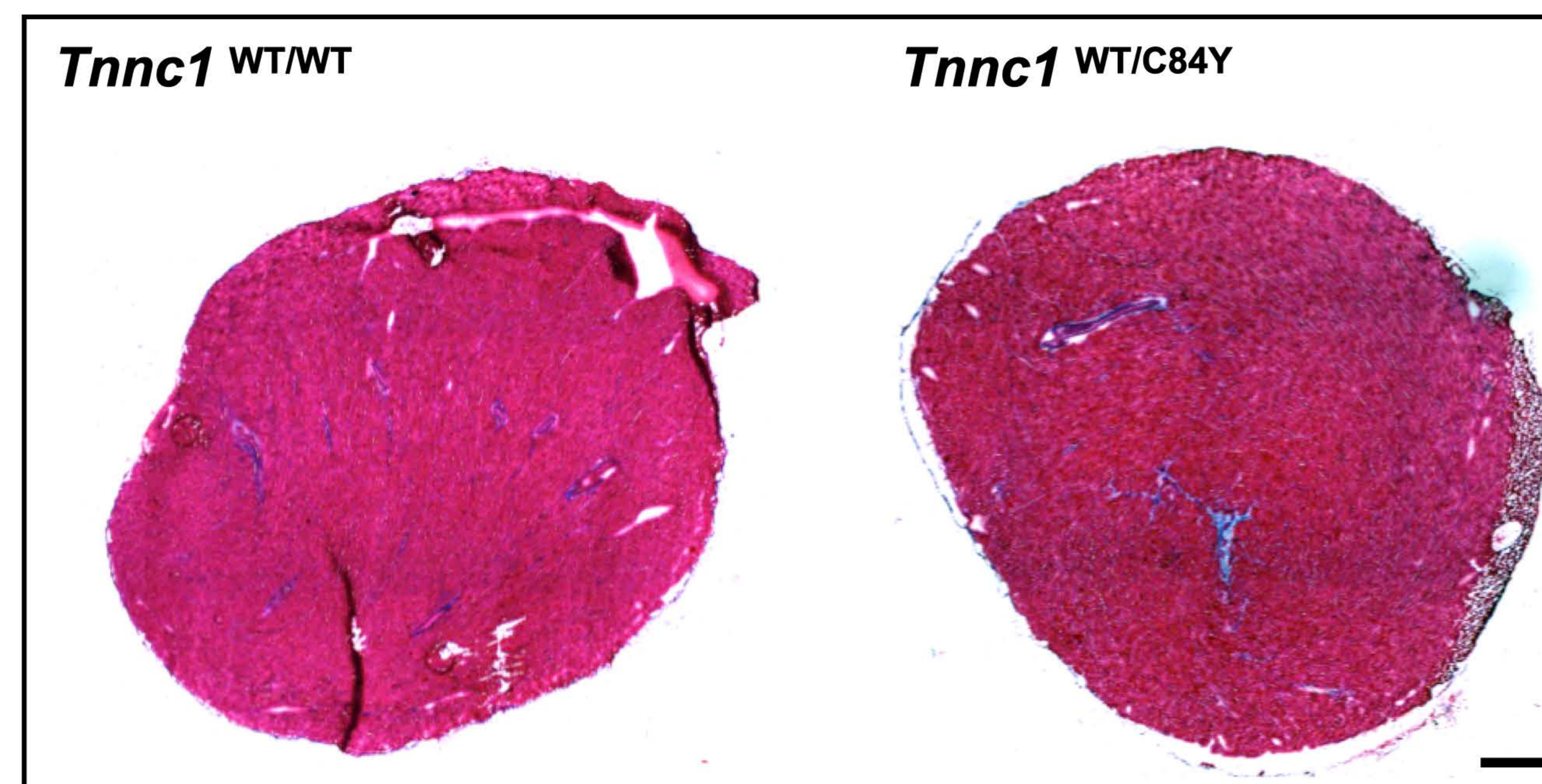

**c**

*Tnnc1* WT/WT

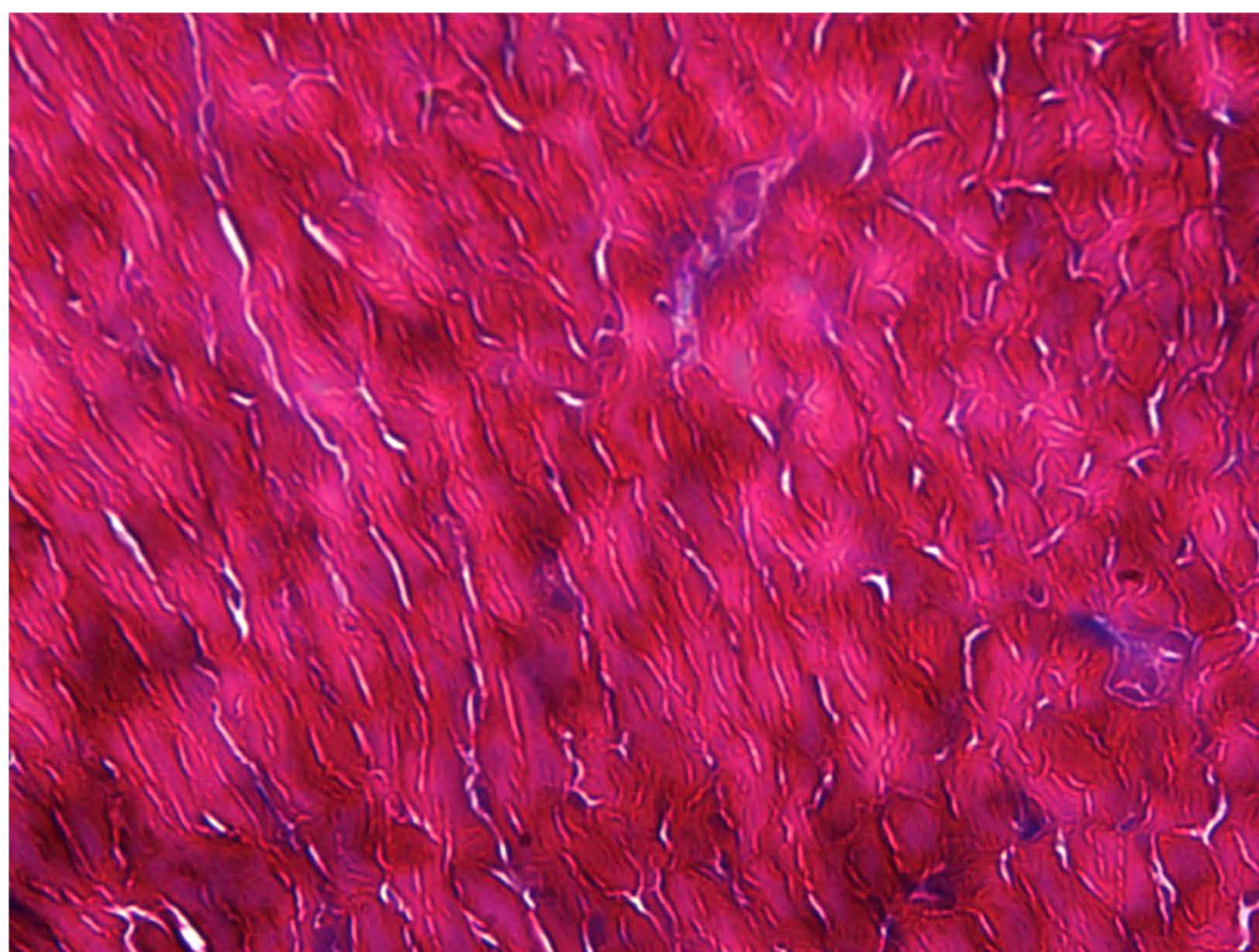

*Tnnc1* WT/C84Y

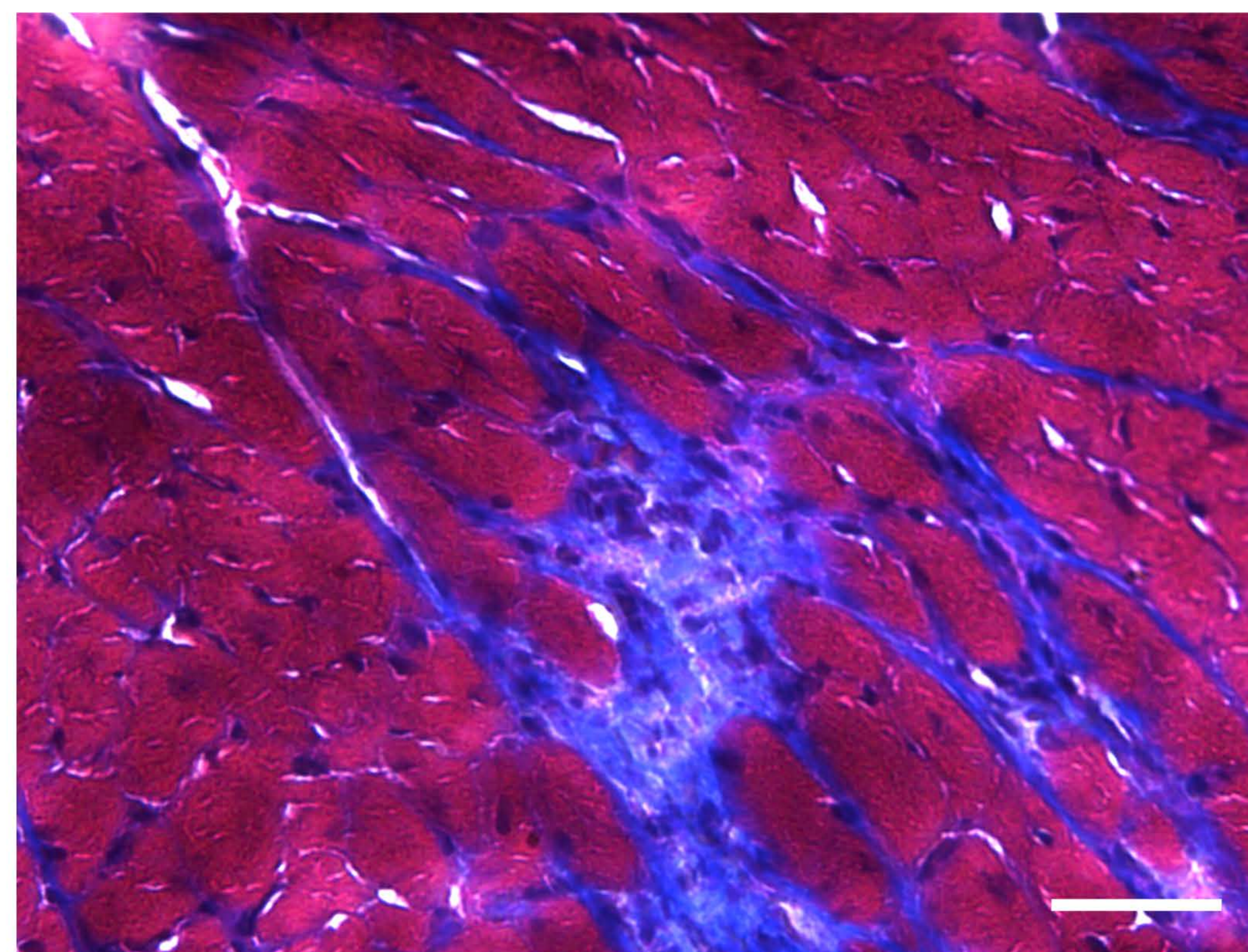

Supplement: SC-012-D1SC01886H-s013 [file SC-012-D1SC01886H-s013.pdf]

**a**

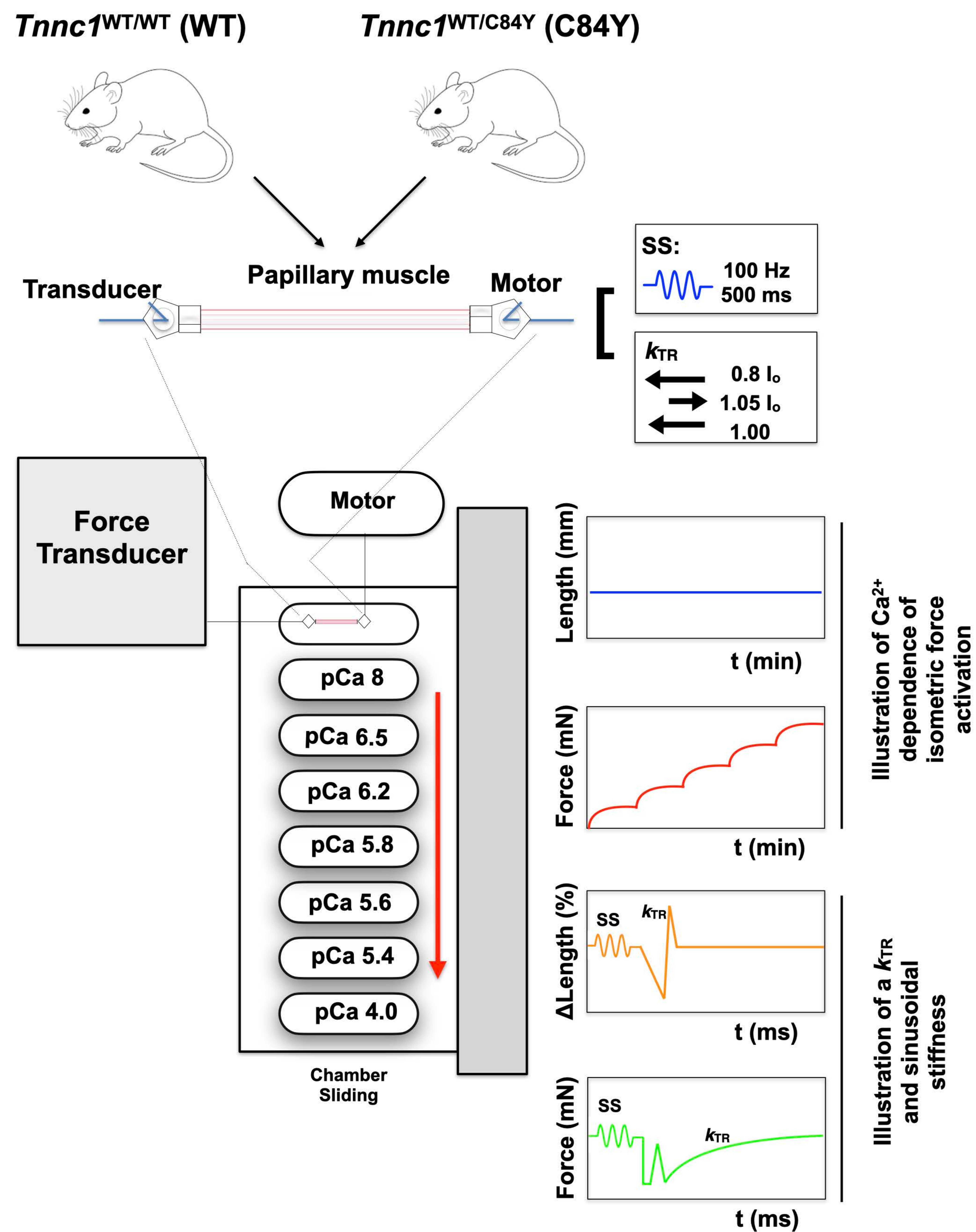

**b**

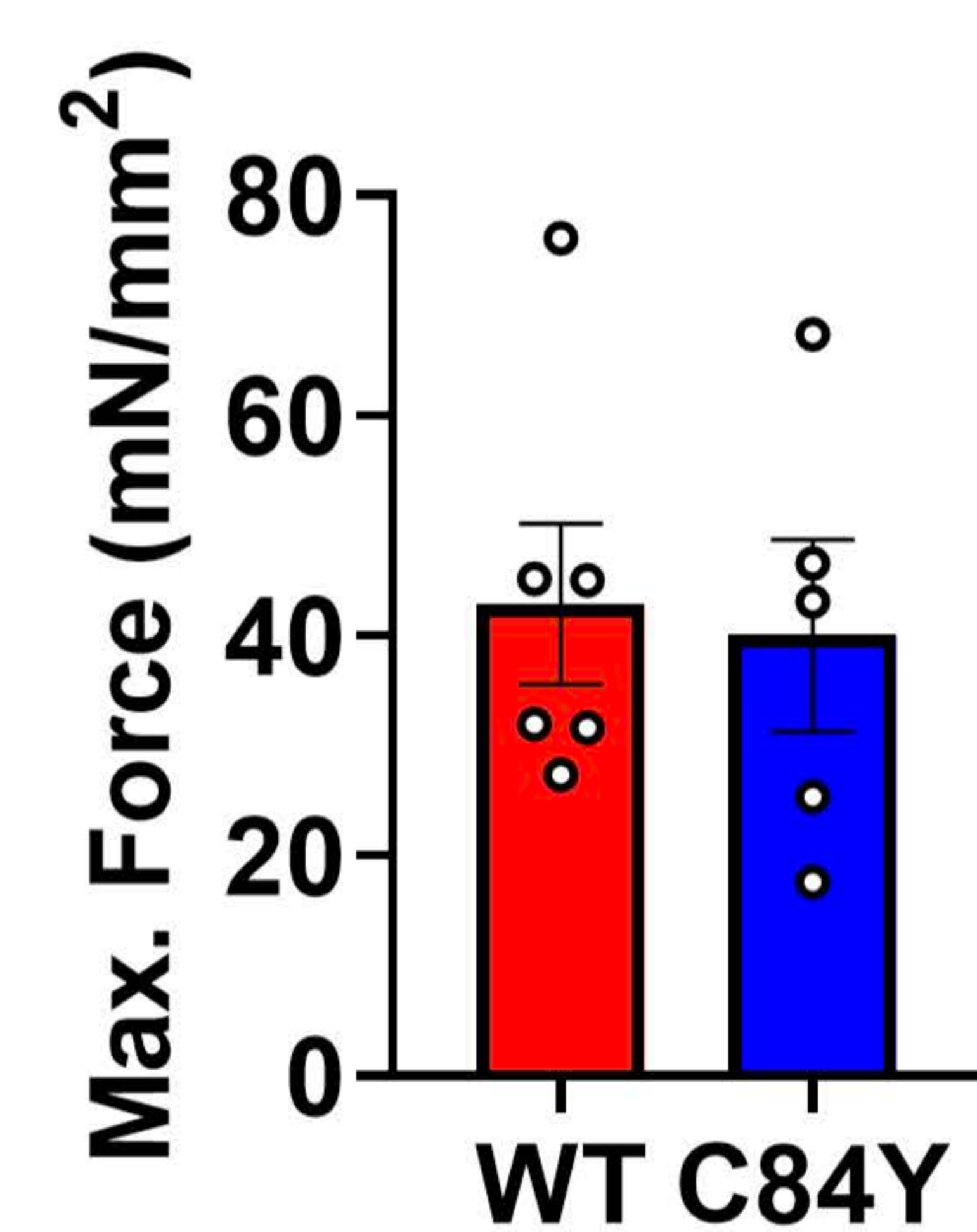

**f**

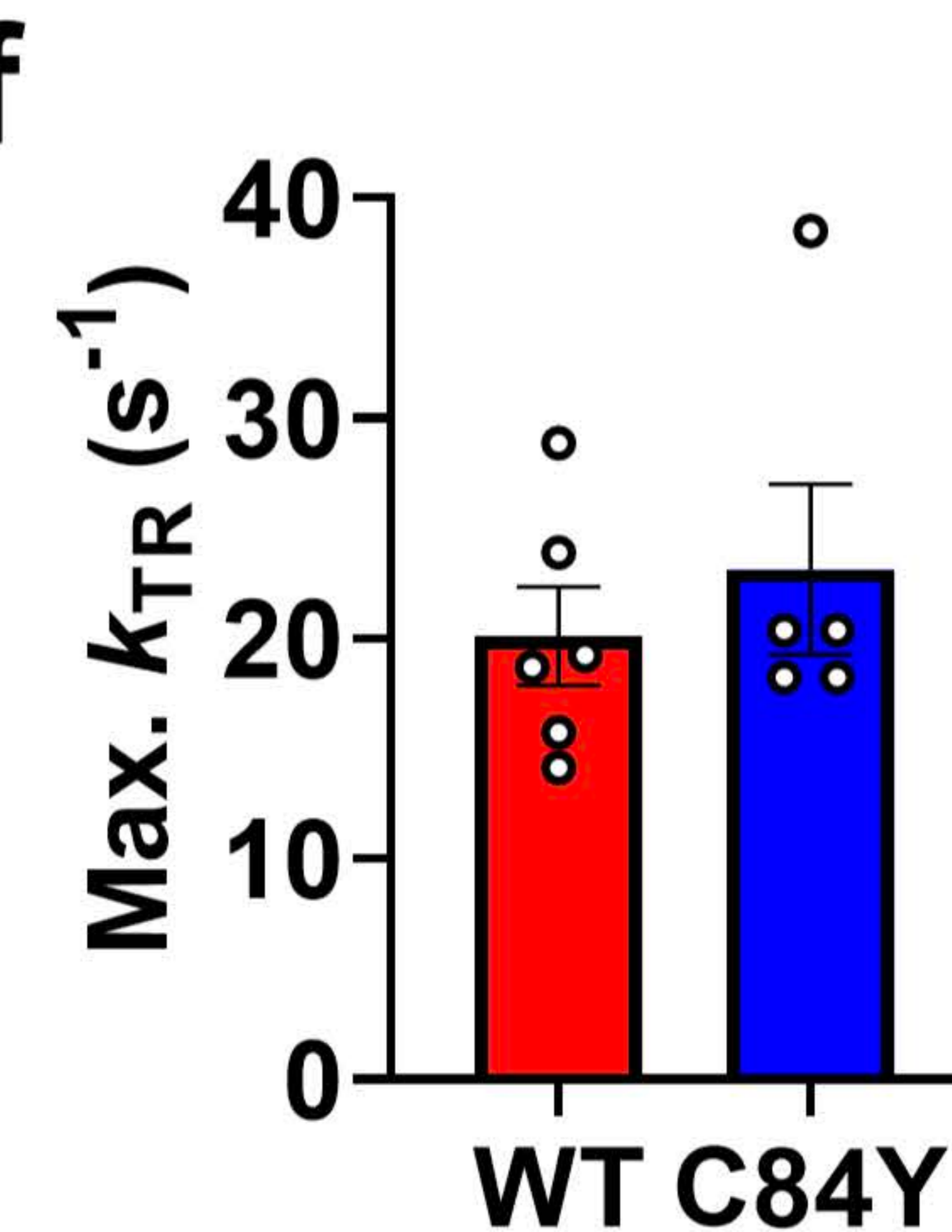

**c**

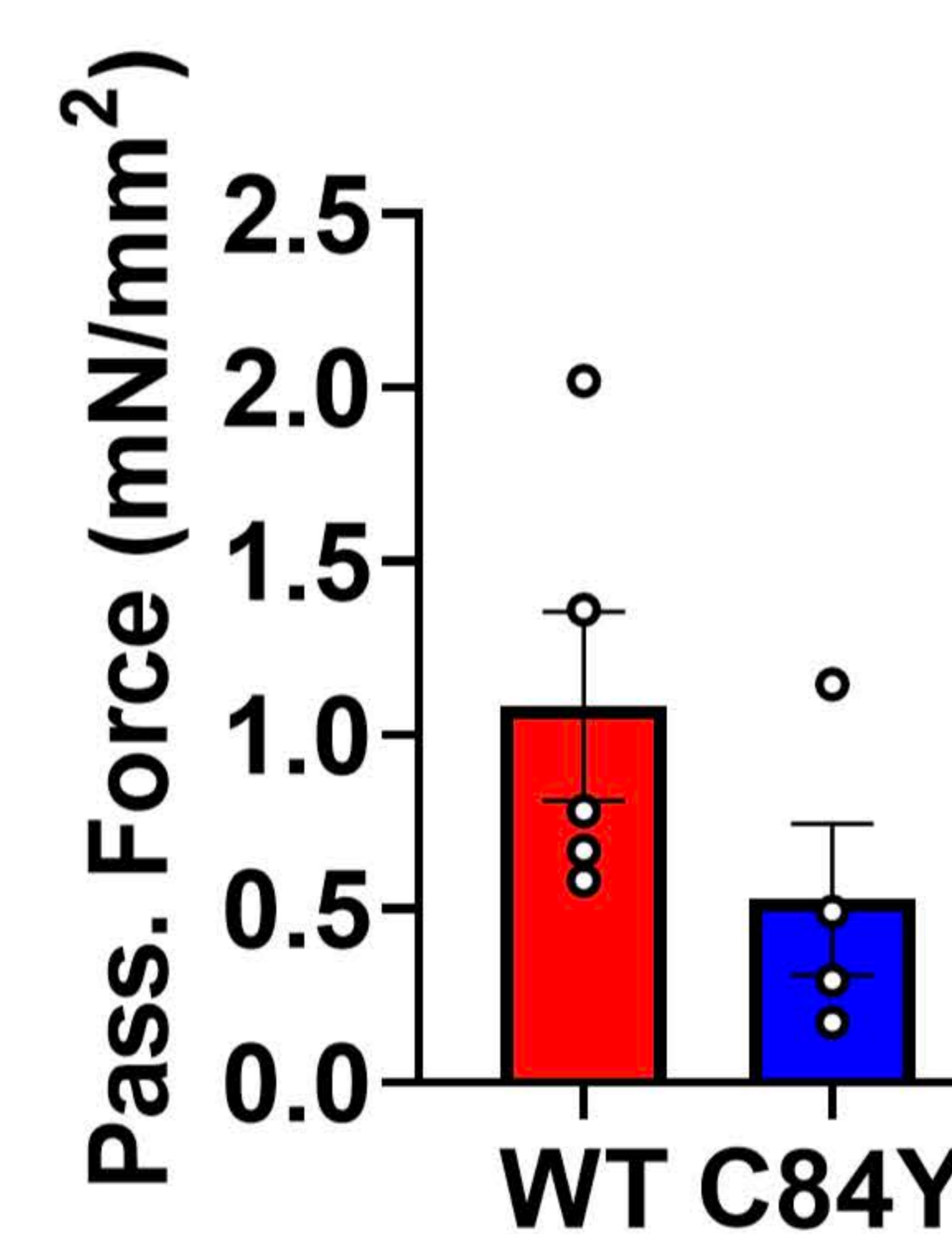

**g**

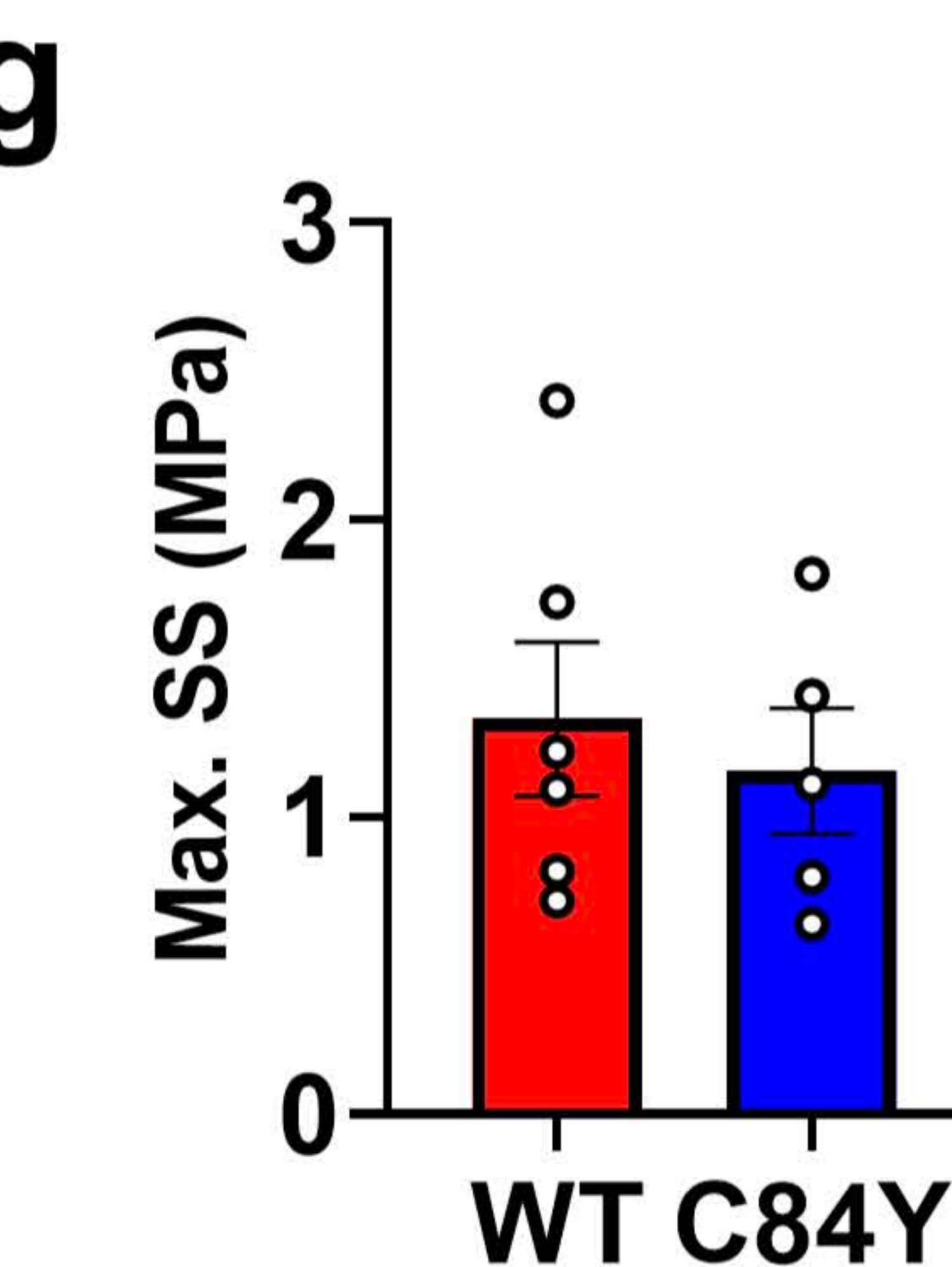

**d**

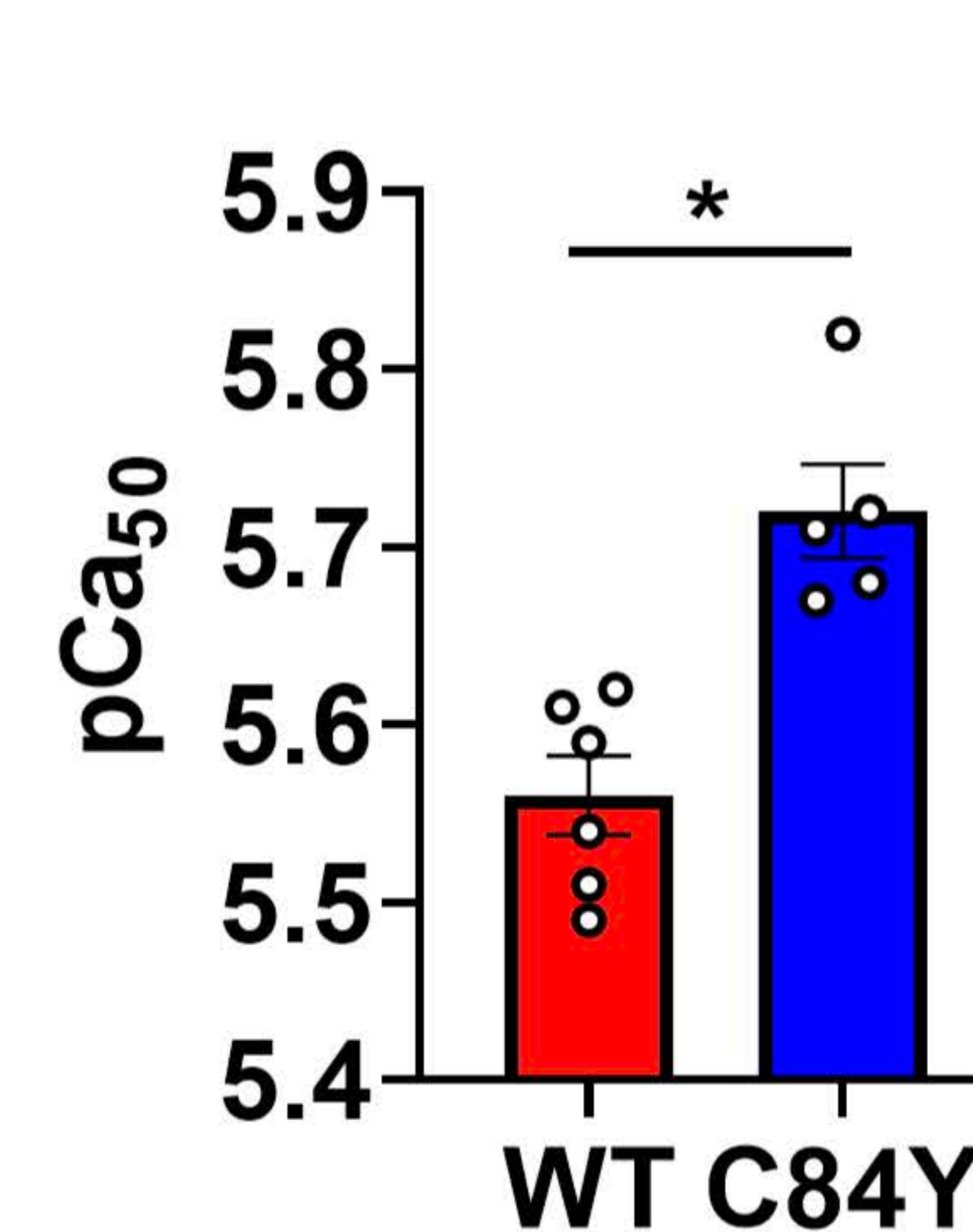

**h**

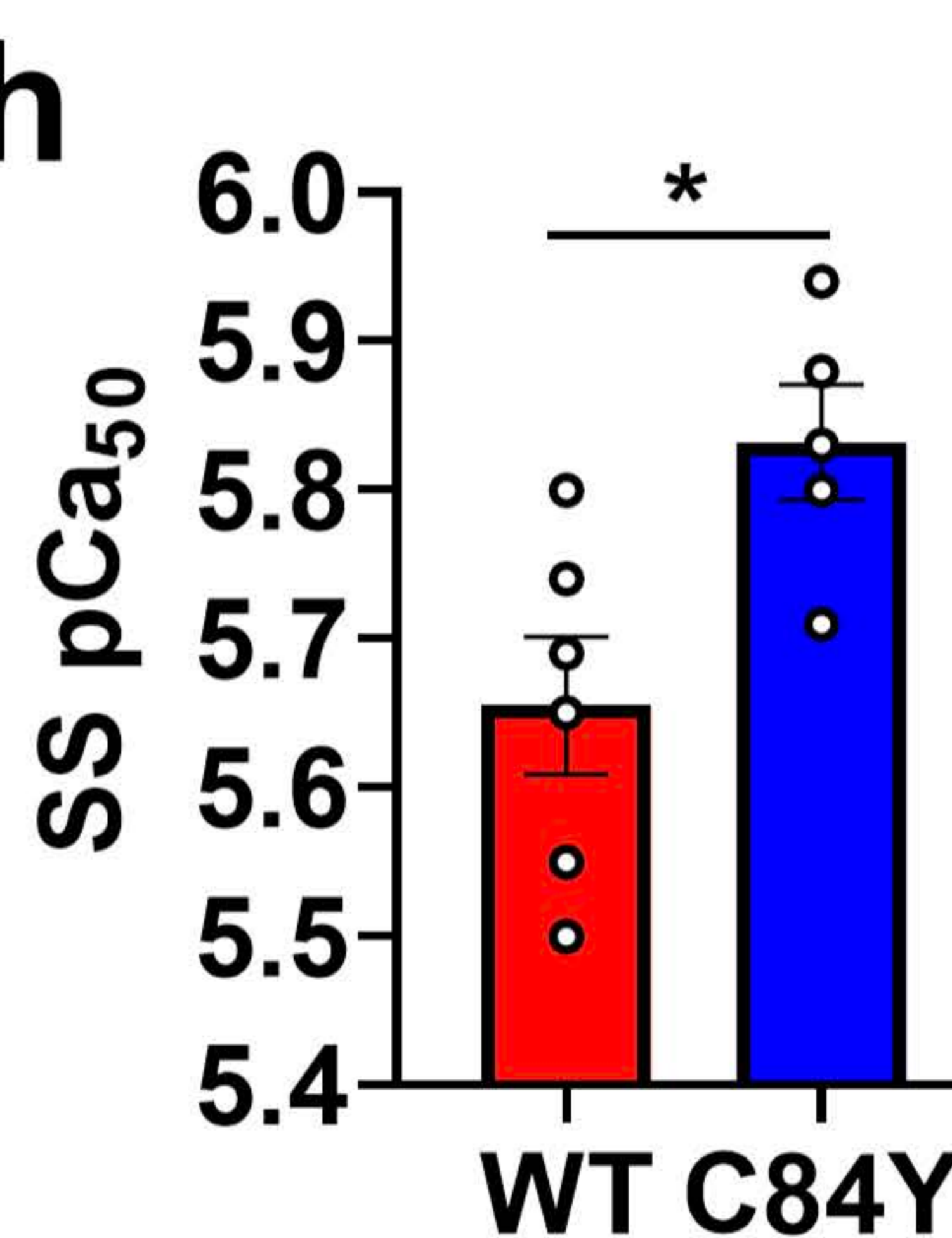

**e**

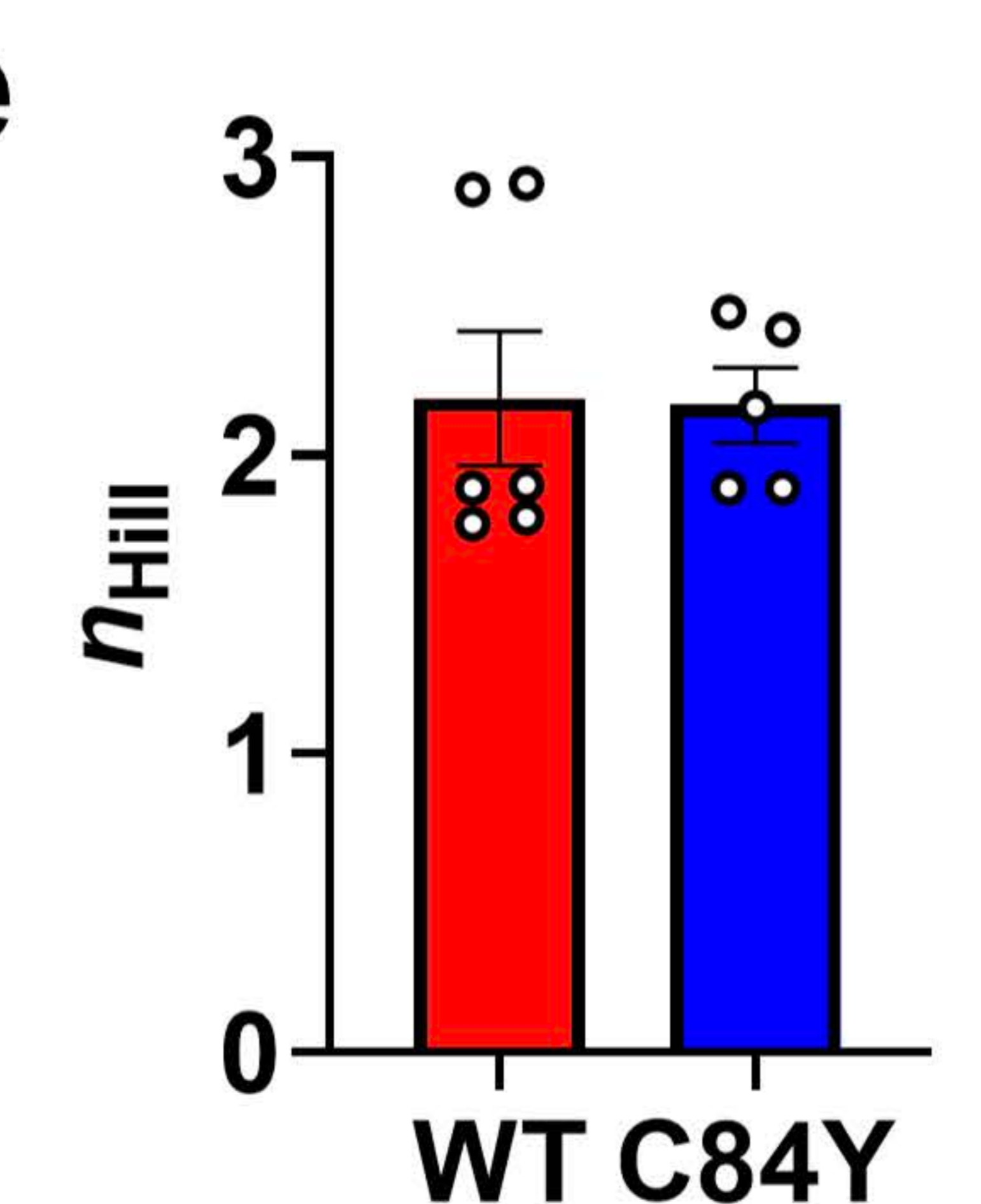

**i**

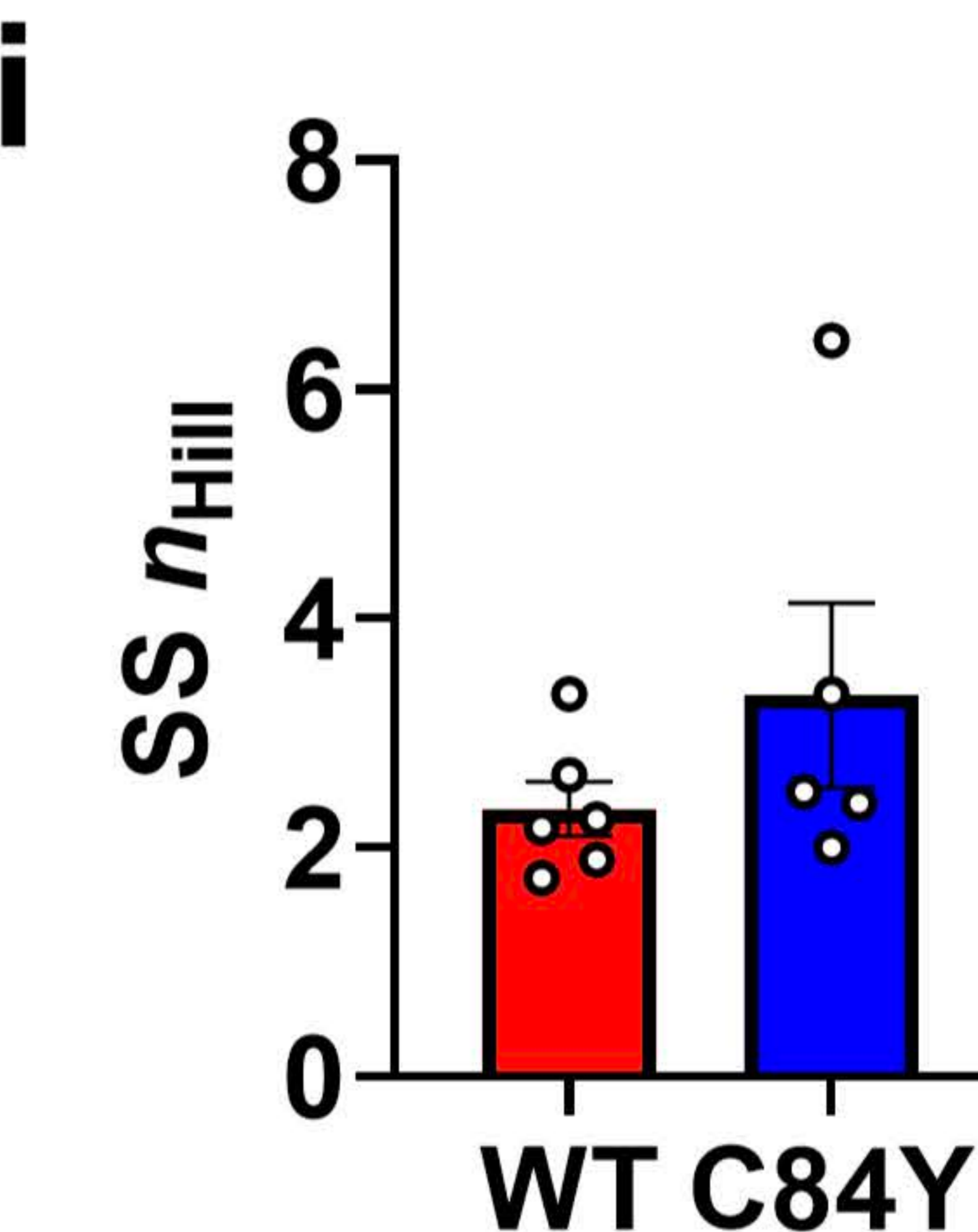

Supplement: SC-012-D1SC01886H-s014 [file SC-012-D1SC01886H-s014.pdf]

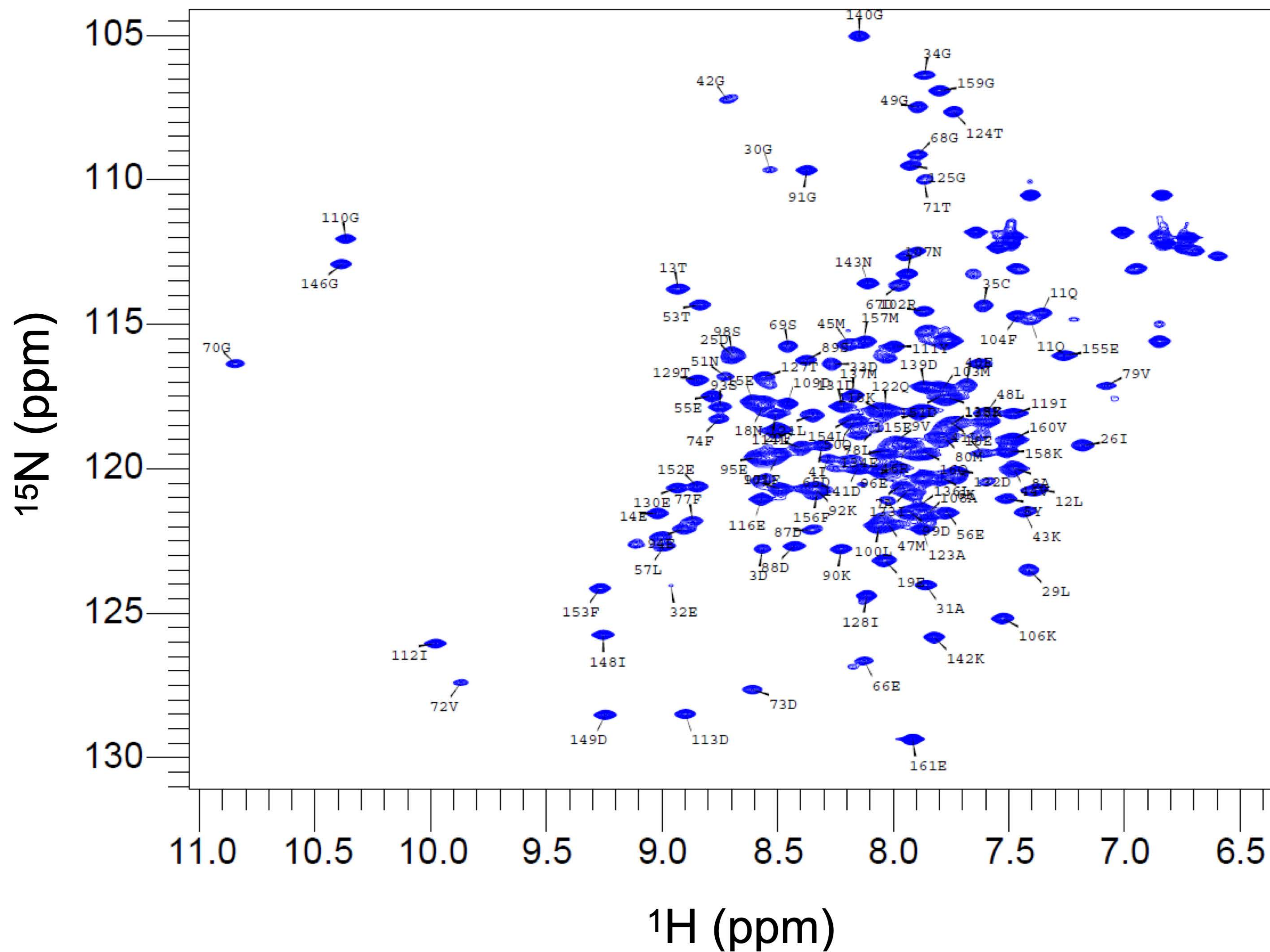

Supplement: SC-012-D1SC01886H-s015 [file SC-012-D1SC01886H-s015.pdf]

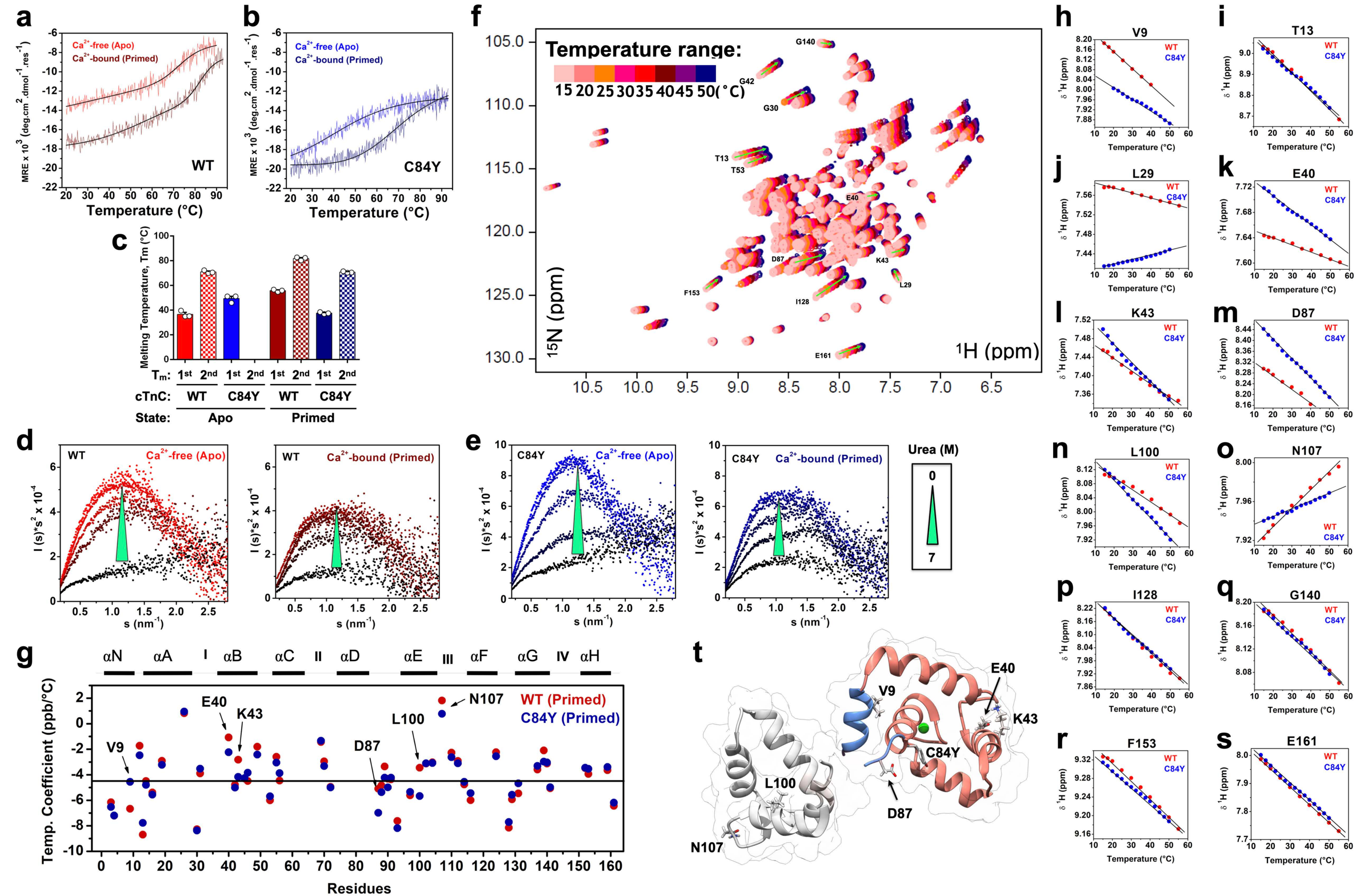

Supplement: SC-012-D1SC01886H-s016 [file SC-012-D1SC01886H-s016.pdf]

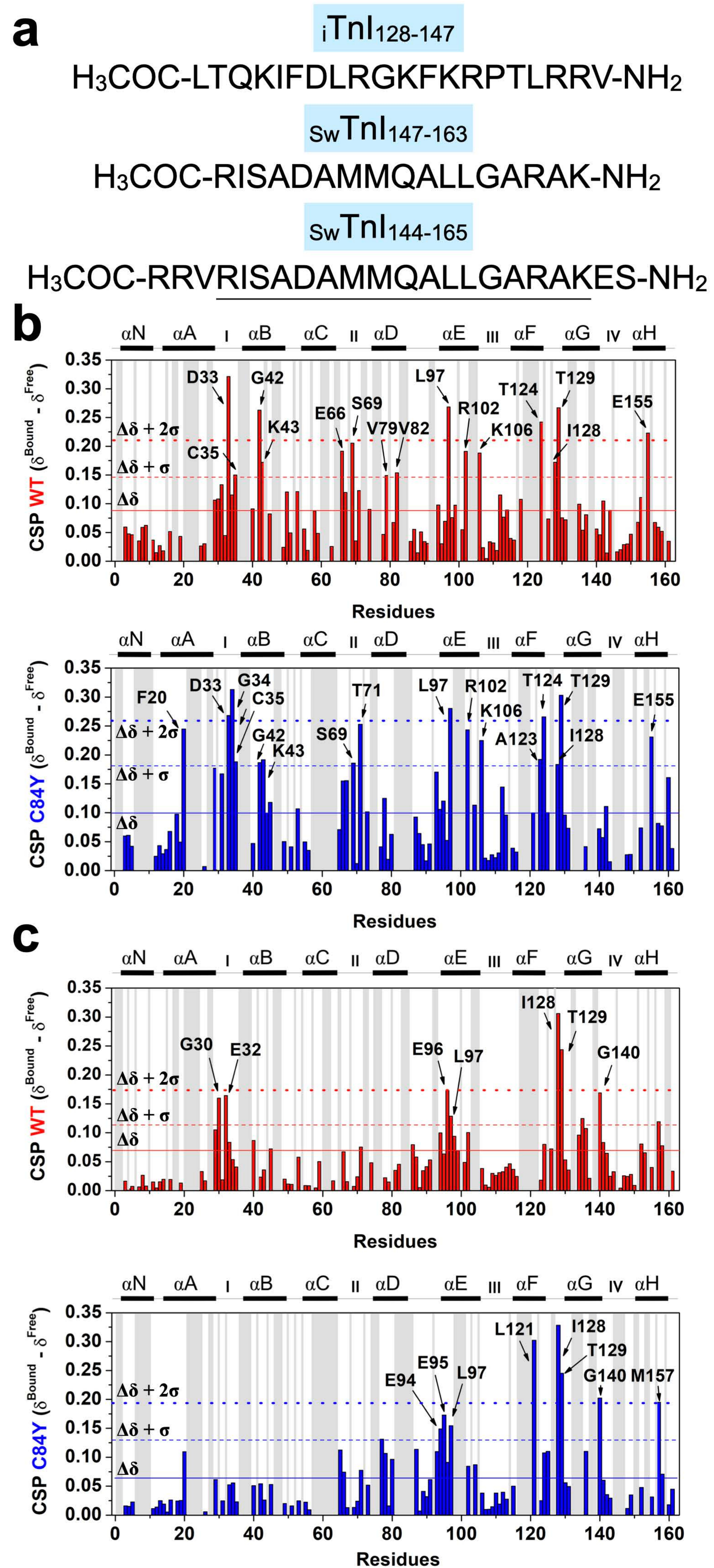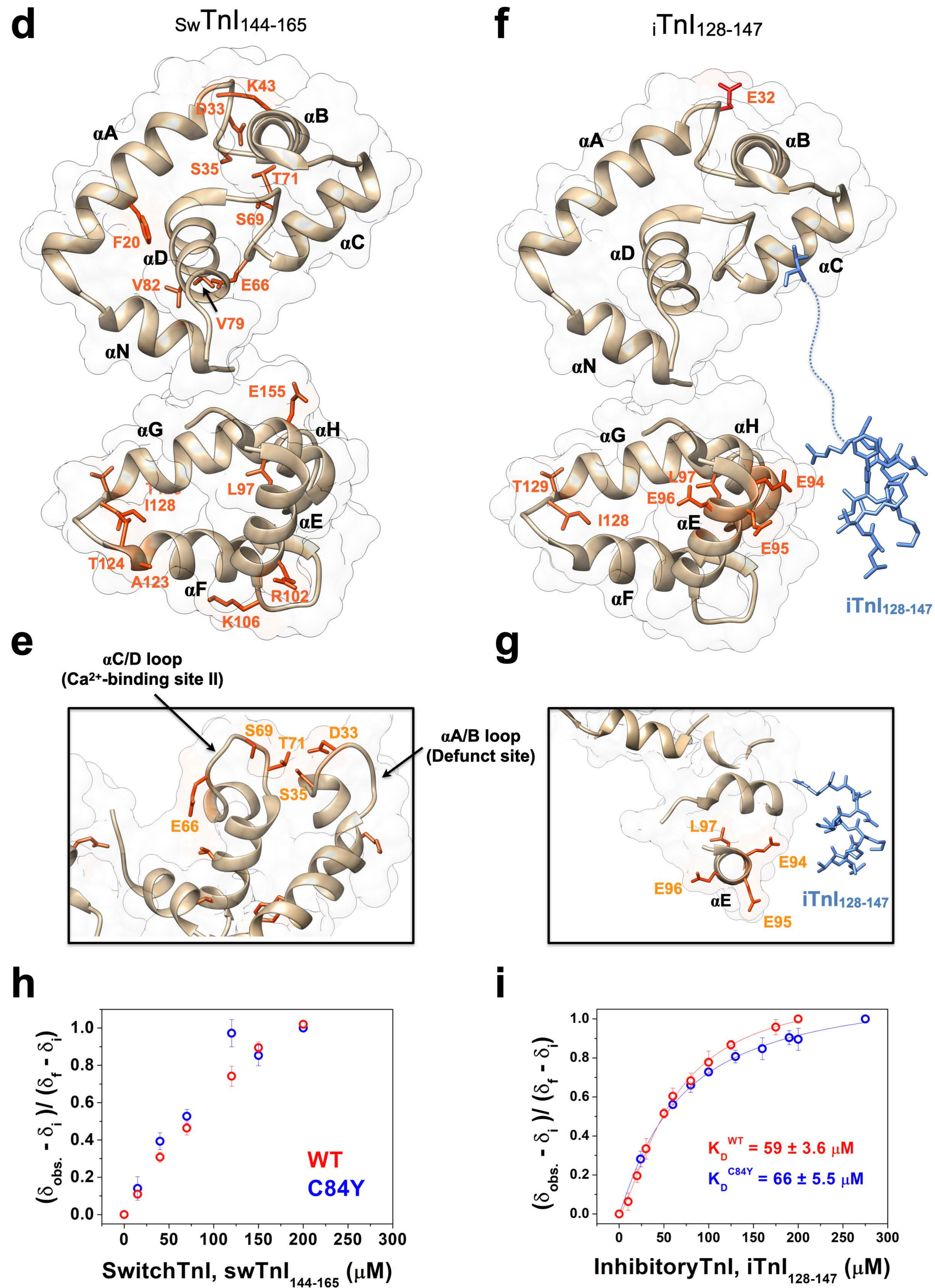

Supplement: SC-012-D1SC01886H-s017 [file SC-012-D1SC01886H-s017.pdf]
