# Supplementary material for: Anomalous structural dynamics of minimally frustrated residues in cardiac troponin C triggers hypertrophic cardiomyopathy
Source: Chem Sci. 2021 Apr 29;12(21):7308–23. doi: 10.1039/d1sc01886h (PMC8171346; doi:10.1039/d1sc01886h)
Supplement: SC-012-D1SC01886H-s007 [file SC-012-D1SC01886H-s007.pdf]

Electronic Supplementary Material (ESI) for Chemical Science.
This journal is © The Royal Society of Chemistry 2021

**Supplementary figures**

**Supplementary Figure 1.** Discrete conformational states of human cardiac TnC N-domain.

**a)** Ribbon representations of the $Ca^{2+}$-free (closed), $Ca^{2+}$-bound (primed), and $Ca^{2+}$/$_{sw}$TnI-bound open active states of cardiac TnC N-domain. β1 and β2 are labeled in the closed state;

**b)** Superposition of the closed and primed states; and

**c)** Superposition of the primed and open active states. The large-scale structural changes of the BC unit are shown as (αB-αB', and αC-αC'). The NAD subunit is negligibly altered. The $_{sw}$TnI peptide is not shown for better presentation of N-TnC structural changes.

**Supplementary Figure 2.** Fluorescence and NMR spectroscopic measurements on human cardiac TnC WT (red) and C84Y (blue).

**a)** bis-ANS fluorescence raw emission spectra of WT cardiac TnC at pCa 8 (red line) and pCa 4 (dark red line);

**b)** bis-ANS fluorescence raw emission spectra of C84Y cardiac TnC at pCa 8 (blue line) and pCa 4 (dark blue line);

**c)** $^{1}H$-$^{15}N$ HSQC spectrum showing assigned $^{1}H$-$^{15}N$ peak correlations for C84Y.

**Supplementary Figure 3.** Temperature- and chemical-induced unfolding of human cardiac TnC WT and C84Y.

**a)** Line plot showing the mean residue ellipticity (MRE) as a function of temperature obtained from CD on WT cardiac TnC in the $Ca^{2+}$-free (apo, or closed state) and $Ca^{2+}$-bound (primed state). Black lines show fitted curves;

**b)** Line plot showing the mean residue ellipticity (MRE) as a function of temperature obtained from circular dichroism data of cTnC C84Y in the $Ca^{2+}$-free (apo, or closed state) or $Ca^{2+}$-bound (primed state). Black lines show fitted curves;

**c)** Dot plot showing melting temperature ($T_m$) values derived from fitted curves of **a** and **b**. First ($1^{st}$) and second ($2^{nd}$) $T_m$ values correspond to temperature-induced unfolding of C-TnC and N-TnC, respectively;

**d and e)** Kratky plots i.e., the intensity (I) of the scattering vector (*s*) multiplied by the scattering vector squared ($s^2$) as a function of the scattering vector of **d,** WT and **e,** C84Y in the $Ca^{2+}$-free (apo) and $Ca^{2+}$-bound (primed) states after treatment with increasing concentrations of urea (1, 3, 5, and 7M);

**f)** $^1H$-$^{15}N$ HSQC spectra collection for C84Y at 5°C temperature increments. Labeling correspond to $^1H$-$^{15}N$ peak correlations presenting systematical chemical shifts to temperature increments.

**g)** Dot plot showing the temperature coefficient dependence as a function of cardiac WT and C84Y TnC residues. Labeled residues correspond to those in which WT and C84Y significantly differed from one another;

**h-s**) Collection of XY plots showing the $^1H$ chemical shift (δ) dependence as a function of temperature increments for several residues illustrate the distinctly temperature influence among same residues in WT and C84Y;

**t)** Labeled residues in **g** are highlighted as sticks within the ribbon representation of cTnC. N-TnC and C-TnC are colored salmon and grey, respectively. αN and the D/E linker are colored blue.

**Supplementary Figure 4.** Switch (sw) and inhibitory (i) cardiac TnI peptide titrations of cTnC WT and C84Y.

**a)** Sequences of peptides used in this study;

**b)** Chemical shift perturbation analysis of bound- against free-peptide ($swTnI_{144-165}$) as a function of WT (red) and C84Y (blue) cardiac TnC residues. Residues deviating from the average values of CSP ($\Delta\delta$) + 1 s.d. ($\Delta\delta + 1\sigma$) and + 2 S.D. ($\Delta\delta + 2\sigma$) are highlighted. Secondary content representation (αN-αH) are depicted as horizontal rectangles at the top. $Ca^{2+}$-binding sites are shown as Roman numerals;

**c)** Chemical shift perturbation analysis of bound- against free-peptide ($iTnI_{128-147}$) as a function of WT (red) and C84Y (blue) cardiac TnC residues. Residues deviating from the average values of CSP ($\Delta\delta$) + 1 S.D. ($\Delta\delta + 1\sigma$) and + 2 S.D. ($\Delta\delta + 2\sigma$) are highlighted. Secondary content representation (αN-αH) are depicted as horizontal rectangles at the top. $Ca^{2+}$-binding sites are shown as Roman numerals;

**d-g)** Labeled residues in **b** and **c** are highlighted as orange sticks within the ribbon representation of cardiac TnC, respectively;

**h, i)** XY plots showing normalized $^1H$ chemical shifts (δ) as a function of the $swTnI_{144-165}$ and the $iTnI_{128-147}$ concentration, respectively.

**Supplementary Figure 5.** $^1H$-$^{15}N$ HSQC spectra regions where variations were observed for WT (red) and C84Y (blue) upon $iTnI_{128-147}$ binding. Black arrows highlight the peak shifts.

**Supplementary Figure 6.** Frustration analysis of evolutionarily diverged TnC isoforms. **a and b)**Tertiary frustration patterns of skeletal and cardiac TnC, respectively, in two different views. Cα contact pairs within a distance of 5 Å are shown as red lines for highly frustrated interactions and green for minimally frustrated pairs; PDB 1TCF (skeletal TnC) and

**c and d)** Contact maps for each studied case in **a** and **b**, respectively. The color scale indicates the local mutational frustration index (see Methods).

**Supplementary Figure 7.** Extreme PCA projections along PC1 and MD simulations with Tn and TF-Tn.

**a)** Representative structures of WT N-TnC near the lower (left) and upper (right) PC1 bounds. The A and B helices are colored black and red, respectively;

**b)** PC1 projection and AB interhelical angle for WT N-TnC (from PDB 1MXL) as a function of time;

**c)** Contact map analysis within TnC. The unit of numbers on color bar is number of average contacts for each pair over the simulation time;

**d)** AB interhelical angles from Tn (PDB 1J1E), TF-Tn $Ca^{2+}$ bound state (PDB 6KN8) and TF-Tn $Ca^{2+}$ free state (6KN7). e) Cα atom RMSF of TnC residues 1-100.

**Supplementary Figure 8.** Dynamics of minimally frustrated segments of human cardiac TnC. Residues in CPMG relaxation dispersion experiments presenting conformational dynamics are highlighted as sticks within the ribbon representation of the cardiac TnC. Black and orange sticks refer to those residues undergoing fast- and intermediate-exchange motions, respectively.

**Supplementary Figure 9.** Generation of *Tnnc1*$^{WT/C84Y}$ knock-in mouse model.

**a** and **b)** Gene-targeting vector strategy used to introduce the C84Y variant into *Tnnc1*;

**c)** Agarose gel electrophoresis of DNA extracted from *Tnnc1*$^{WT/WT}$ and *Tnnc1*$^{WT/C84YNeo}$ mice confirmed the presence of the variant and the NEO cassette (see Methods for further details).

**d)** Crossing of *Tnnc1*$^{WT/C84YNeo}$ with Ella/Cre mice led to NEO cassette excision from *Tnnc1*.

**Supplementary Figure 10.** Masson's trichrome staining was used to determine the amount of fibrosis present in the knock-in *Tnnc1*$^{WT/C84Y}$ hearts.

**a)** The % area of Masson's trichrome stained transverse whole heart sections is compared to total area is reported for WT hearts (n=5) and C84Y heterozygous hearts (n=6). Statistics were performed using the Mann-Whitney test and error shown as S.D. and $*p < 0.05$ was considered significant.

**b)** Transverse heart sections are shown that are stained with Masson's trichrome stain and scale bar = 900 µm.

**c)** Focal fibrotic areas are shown by purple staining of the myocardium in representative 20X images. Bar, 50 µm.

**Supplementary Figure 11.** Illustration of muscle mechanics experiments and experimental data obtained from *Tnnc1*$^{WT/WT}$ and *Tnnc1*$^{WT/C84Y}$ knock-in mice.

**a**) Illustration of the muscle mechanics experiments;

**b-i**) Quantitative comparison of contractile parameters obtained from *Tnnc1*$^{WT/WT}$ (WT, red) and *Tnnc1*$^{WT/C84Y}$ (C84Y, blue) CMPs:

**b**) Max. Force = maximum steady-state isometric force at saturating $Ca^{2+}$;

**c**) Pass. Force = minimum steady-state isometric force at low $Ca^{2+}$;

**d**) $p\text{Ca}_{50}$ = $Ca^{2+}$ concentration needed to reach 50% of the maximum steady-state isometric force;

**e**) $n_{\text{Hill}}$ = Hill coefficient which is the slope of the force-pCa relation around $p\text{Ca}_{50}$, and is indicative of cooperativity of thin filament activation;

**f**) Max. $k_{\text{TR}}$ = maximum rate of tension redevelopment at saturating $Ca^{2+}$;

**g**) Max. SS = maximum steady-state sinusoidal stiffness at saturating $Ca^{2+}$;

**h**) $SS_{p\text{Ca}50}$ = $Ca^{2+}$ concentration needed to reach 50% of the maximum steady-state sinusoidal stiffness;

**i**) $SS_{nHill}$ = Hill coefficient which is the slope of the SS-pCa relation around $SS_{p\text{Ca}50}$, and is indicative of cooperative $Ca^{2+}$-activation of sinusoidal stiffness.

Horizontal bars represent mean values. Statistical significance was tested using unpaired Student's *t*-test * $p < 0.02$.

**Supplementary Table 1**: Echocardiographic parameters of *Tnnc1*$^{WT/WT}$ (WT) and *Tnnc1*$^{WT/C84Y}$ knock-in mice.

| **Echo** | **3 months** | | **6 months** | |
|---|---|---|---|---|
| **Parameter** | **WT (*n* = 5)** | **C84Y (*n* = 7)** | **WT (*n* = 5)** | **C84Y (*n* = 6)** |
| **Male** | **2** | **4** | **2** | **4** |
| **Female** | **3** | **3** | **3** | **2** |
| AET (ms) | 44.4±0.6 | 51.1±2.9 | 46.4±2.0 | 51.6±1.8 |
| IVCT (ms) | 13.9±1.8 | 28.1±3.7* | 20.4±2.1 | 20.1±4.0 |
| IVRT (ms) | 21.0±2.4 | 25.0±4.0 | 15.0±2.4 | 22.5±1.1* |
| MV A (mm/s) | 492.6±27.2 | 562.2±50.5 | 482.3±32.7 | 523.1±39.8 |
| MV Decel (mm/s2) | -95574.3±22164.7 | -61082.1±9733.3 | -75645.5±19507.7 | -73708.8±28032.0 |
| MV Decel (ms) | 9.9±2.3 | 12.3±1.8 | 10.9±2.3 | 13.0±2.8 |
| MV PHT (ms) | 10.6±0.8 | 10.3±1.0 | 10.1±0.8 | 11.5±0.9 |
| NFT (ms) | 79.2±1.4 | 103.7±5.1* | 81.9±3.2 | 94.4±4.2 |
| MV E/A | 1.5±0.0 | 1.1±0.1* | 1.4±0.1 | 1.2±0.1 |
| Heart Rate (BPM) | 504.0±7.4 | 450.0±11.6* | 500.9±4.7 | 451.6±12.7* |
| LVAW;d (mm) | 1.2±0.1 | 1.3±0.1 | 1.1±0.0 | 1.3±0.1 |
| LVAW;s (mm) | 1.5±0.1 | 1.6±0.1 | 1.5±0.1 | 1.7±0.1 |
| LVPW;d (mm) | 1.0±0.1 | 1.0±0.1 | 0.8±0.1 | 1.2±0.1* |
| LVPW;s (mm) | 1.2±0.1 | 1.3±0.0 | 1.1±0.1 | 1.5±0.0* |
| CO (mL/min) | 20.1±1.7 | 13.6±1.5* | 22.4±1.9 | 15.3±1.0* |
| Diameter;d (mm) | 4.2±0.1 | 3.6±0.2* | 4.2±0.1 | 3.7±0.1* |
| Diameter;s (mm) | 3.2±0.1 | 2.6±0.2 | 3.0±0.1 | 2.6±0.2 |
| EF (%) | 49.9±3.3 | 56.8±5.1 | 56.1±4.1 | 58.7±4.1 |
| FS (%) | 25.2±2.0 | 29.9±3.7 | 29.3±2.6 | 30.8±2.9 |
| SV (uL) | 39.9±3.4 | 30.9±2.8 | 45.0±3.7 | 34.0±2.1* |
| V;d (uL) | 79.9±5.0 | 56.6±6.6* | 80.3±3.1 | 58.7±4.1* |
| V;s (uL) | 40.0±3.5 | 25.6±5.4 | 35.3±3.8 | 24.8±3.3 |

Data are presented as AVG ± S.E.M. AET = aortic ejection time; IVCT = isovolumetric contraction time; IVRT = isovolumetric relaxation time; MV A = mitral valve atrial-peak-flow velocity; MV Decel = mitral valve deceleration; MV Decel = mitral valve deceleration time; MV PHT = mitral valve pressure half time; NFT = no flow time; MV E/A = mitral valve early-peak-flow to atrial-peak-flow velocity ratio; LVAW;d/s = diastolic/systolic left ventricular anterior wall thickness; LVPW;d/s = diastolic/systolic left ventricular posterior wall thickness; CO = cardiac output; Diameter;d/s = diastolic/systolic diameter; EF = ejection fraction; FS = fractional shortening; SV = stroke volume; V;d/s = diastolic/systolic diameter; Statistical significance was determined by using One-way ANOVA with post-hoc Student-Newman-Keuls test * $p < 0.05$.

**Supplementary Table 2.** Values of contractile parameters exhibited by *Tnnc1*$^{WT/WT}$ (WT) and *Tnnc1*$^{WT/C84Y}$ (C84Y) CMPs shown in Figure 6.

| | WT | C84Y |
|---|---|---|
| **$F_{max}$ (mN/mm$^2$)** | 42.86 ± 7.30 | 40.00 ± 8.70 |
| **$F_{passive}$ (mN/mm$^2$)** | 01.08 ± 0.27 | 00.53 ± 0.22 |
| **$FpCa_{50}$** | 05.56 ± 0.02 | 05.72 ± 0.02* |
| **$n_{Hill}$** | 02.19 ± 0.22 | 02.16 ± 0.12 |
| **$k_{TRmax}$ (s$^{-1}$)** | 20.10 ± 2.22 | 23.14 ± 3.86 |
| **$SS_{max}$ (MPa)** | 01.33 ± 0.25 | 01.15 ± 0.21 |
| **$SS_{pCa50}$** | 05.65 ± 0.04 | 05.83 ± 0.03* |
| **$SSn_{Hill}$** | 02.34 ± 0.24 | 03.32 ± 0.80 |
| **#CMP** | 6 | 5 |

$F_{max}$ = maximum steady-state isometric force; $F_{passive}$ = minimum steady-state isometric force; $FpCa_{50}$ = $Ca^{2+}$ concentration needed to reach 50% of the maximum steady-state isometric force; $n_{Hill}$ = cooperativity of thin filament activation; $k_{TR\,max}$ = maximum rate of tension redevelopment; $SS_{max}$ = maximum steady-state sinusoidal stiffness; $SS_{pCa50}$ = $Ca^{2+}$ concentration needed to reach 50% of the maximum steady-state sinusoidal stiffness.

*$p = 0.001$ from unpaired Student's *t*-test comparing C84Y $pCa_{50}$ vs. WT $pCa_{50}$.

*$p = 0.019$ from unpaired Student's *t*-test comparing C84Y $SSpCa_{50}$ vs. WT $SSpCa_{50}$.

**Supplementary Table 3.** Optimized parameter estimates and predictions from the 3-state model for force-$k_{TR}$ relationships shown in Figure 6d.

| | **3-state model fitted parameters** | | | | **3-state model predictions** | | |
|---|---|---|---|---|---|---|---|
| | ***f*** **($s^{-1}$)** | ***g*** **($s^{-1}$)** | **$k_{ON}$ ($M^{-1}$ $s^{-1}$)** | **$k_{OFF}$ ($s^{-1}$)** | **F*p*$Ca_{50}$** | **$F_{max\ (norm)}$** | **$k_{TR\ max}$ ($s^{-1}$)** |
| *Tnnc1*$^{WT/WT}$ | 8.19 | 12.39 | 1.84 x $10^8$ | 890.68 | 5.56 | 1.00 | 20.06 |
| *Tnnc1*$^{WT/C84Y}$ | 18.86 | 5.75 | 1.84 x $10^8$ | 1558.11 | 5.72 | 0.93 | 23.14 |

*f* = cross-bridge attachment rate; *g* = cross-bridge detachment rate; $k_{ON}$ = 2nd order rate constant for $Ca^{2+}$ association; $k_{OFF}$ = rate constant for $Ca^{2+}$ dissociation; F*p*$Ca_{50}$ = $Ca^{2+}$ concentration needed to reach 50% of the maximum steady-state isometric force; $F_{max\ (norm)}$ = maximum steady-state isometric force normalized to WT; $k_{TR\ max}$ = maximum rate of tension redevelopment.
