# Supplementary material for: Anomalous structural dynamics of minimally frustrated residues in cardiac troponin C triggers hypertrophic cardiomyopathy
Source: Chem Sci. 2021 Apr 29;12(21):7308–23. doi: 10.1039/d1sc01886h (PMC8171346; doi:10.1039/d1sc01886h)
Supplement: SC-012-D1SC01886H-s008 [file SC-012-D1SC01886H-s008.pdf]

Electronic Supplementary Material (ESI) for Chemical Science.
This journal is © The Royal Society of Chemistry 2021

Supplementary Table 1: Echocardiographic parameter of *Tnnc1*$^{WT/WT}$ (WT) and *Tnnc1*$^{WT/C84Y}$ (C84Y) knock-in mice.

**Echo**	**3 months**		**6 months**	
**Parameter**	**WT (n=5)**	**C84Y (n=7)**	**WT (n=5)**	**C84Y (n=6)**
**Male**	**2**	**4**	**2**	**4**
**Female**	**3**	**3**	**3**	**2**
AET (ms)	44.4±0.6	51.1±2.9	46.4±2.0	51.6±1.8
IVCT (ms)	13.9±1.8	28.1±3.7*	20.4±2.1	20.1±4.0
IVRT (ms)	21.0±2.4	25.0±4.0	15.0±2.4	22.5±1.1*
MV A (mm/s)	492.6±27.2	562.2±50.5	482.3±32.7	523.1±39.8
MV Decel (mm/s2)	-95574.3±22164.7	-61082.1±9733.3	-75645.5±19507.7	-73708.8±28032.0
MV Decel (ms)	9.9±2.3	12.3±1.8	10.9±2.3	13.0±2.8
MV PHT (ms)	10.6±0.8	10.3±1.0	10.1±0.8	11.5±0.9
NFT (ms)	79.2±1.4	103.7±5.1*	81.9±3.2	94.4±4.2
MV E/A	1.5±0.0	1.1±0.1*	1.4±0.1	1.2±0.1
Heart Rate (BPM)	504.0±7.4	450.0±11.6*	500.9±4.7	451.6±12.7*
LVAW;d (mm)	1.2±0.1	1.3±0.1	1.1±0.0	1.3±0.1
LVAW;s (mm)	1.5±0.1	1.6±0.1	1.5±0.1	1.7±0.1
LVPW;d (mm)	1.0±0.1	1.0±0.1	0.8±0.1	1.2±0.1*
LVPW;s (mm)	1.2±0.1	1.3±0.0	1.1±0.1	1.5±0.0*
CO (mL/min)	20.1±1.7	13.6±1.5*	22.4±1.9	15.3±1.0*
Diameter;d (mm)	4.2±0.1	3.6±0.2*	4.2±0.1	3.7±0.1*
Diameter;s (mm)	3.2±0.1	2.6±0.2	3.0±0.1	2.6±0.2
EF (%)	49.9±3.3	56.8±5.1	56.1±4.1	58.7±4.1
FS (%)	25.2±2.0	29.9±3.7	29.3±2.6	30.8±2.9
LV Mass Corr (mg)	157.8±10.5	134.1±10.7	131.3±10.2	158.7±14.4
SV (uL)	39.9±3.4	30.9±2.8	45.0±3.7	34.0±2.1*
V;d (uL)	79.9±5.0	56.6±6.6*	80.3±3.1	58.7±4.1*
V;s (uL)	40.0±3.5	25.6±5.4	35.3±3.8	24.8±3.3
