# Supplementary material for: Anomalous structural dynamics of minimally frustrated residues in cardiac troponin C triggers hypertrophic cardiomyopathy
Source: Chem Sci. 2021 Apr 29;12(21):7308–23. doi: 10.1039/d1sc01886h (PMC8171346; doi:10.1039/d1sc01886h)
Supplement: SC-012-D1SC01886H-s009 [file SC-012-D1SC01886H-s009.pdf]

Electronic Supplementary Material (ESI) for Chemical Science.
This journal is © The Royal Society of Chemistry 2021

Supplementary Table 2. Values of Contractile Parameters Exhibited by *Tnnc1*$^{WT/WT}$ (WT) and *Tnnc1*$^{WT/C84Y}$ (C84Y) CMPs.

	WT	C84Y
**F_{max} (mN/mm^2)**	42.86 ± 7.30	40.00 ± 8.70
**$F_{passive}$ (mN/mm^2)**	01.08 ± 0.27	00.53 ± 0.22
**pCa_{50}**	05.56 ± 0.02	05.72 ± 0.02*
**n_{Hill}**	02.19 ± 0.22	02.16 ± 0.12
**$k_{TR\ max}$ (s^{-1})**	20.10 ± 2.22	23.14 ± 3.86
**SS_{max} (MPa)**	01.33 ± 0.25	01.15 ± 0.21
**SS_{pCa50}**	05.65 ± 0.04	05.83 ± 0.03*
**SSn_{Hill}**	02.34 ± 0.24	03.32 ± 0.80
**#CMP**	6	5

*p=0.001 from unpaired t-test comparing C84Y pCa_{50} vs. WT pCa_{50}.
*p=0.019 from unpaired t-test comparing C84Y $SSpCa_{50}$ vs. WT $SSpCa_{50}$.

Supplementary Table 3. Optimized parameter estimates and predictions from the 3-state model for force-k_{TR} data depicted in Figure 6.

**Reconstituted CMP**	**3-state model fitted parameters**				**3-state model predictions**		
	f **(s^{-1})**	***g*** **(s^{-1})**	**k_{ON} (M^{-1} s^{-1})**	**k_{OFF} (s^{-1})**	**pCa_{50}**	**max force (norm)**	**$k_{TR\ max}$ (s^{-1})**
WT	8.19	12.39	1.84×10^8	890.67	5.56	1.00	20.10
C84Y	26.54	5.76	1.34×10^8	2040.96	5.72	0.93	23.14
